# Diagnostic performance of radiomics in adrenal masses: A systematic review and meta-analysis

**DOI:** 10.3389/fonc.2022.975183

**Published:** 2022-09-02

**Authors:** Hao Zhang, Hanqi Lei, Jun Pang

**Affiliations:** Department of Urology, Kidney and Urology Center, Pelvic Floor Disorders Center, The Seventh Affiliated Hospital, Sun Yat-sen University, Shenzhen, China

**Keywords:** adrenal tumor, radiomics, machine learning, diagnostic performance, radiomics quality score

## Abstract

**Objectives:**

(1) To assess the methodological quality and risk of bias of radiomics studies investigating the diagnostic performance in adrenal masses and (2) to determine the potential diagnostic value of radiomics in adrenal tumors by quantitative analysis.

**Methods:**

PubMed, Embase, Web of Science, and Cochrane Library databases were searched for eligible literature. Methodological quality and risk of bias in the included studies were assessed by the Quality Assessment of Diagnostic Accuracy Studies 2 (QUADAS-2) and Radiomics Quality Score (RQS). The diagnostic performance was evaluated by pooled sensitivity, specificity, diagnostic odds ratio (DOR), and area under the curve (AUC). Spearman’s correlation coefficient and subgroup analysis were used to investigate the cause of heterogeneity. Publication bias was examined using the Deeks’ funnel plot.

**Results:**

Twenty-eight studies investigating the diagnostic performance of radiomics in adrenal tumors were identified, with a total of 3579 samples. The average RQS was 5.11 (14.2% of total) with an acceptable inter-rater agreement (ICC 0.94, 95% CI 0.93–0.95). The risk of bias was moderate according to the result of QUADAS-2. Nine studies investigating the use of CT-based radiomics in differentiating malignant from benign adrenal tumors were included in the quantitative analysis. The pooled sensitivity, specificity, DOR and AUC with 95% confidence intervals were 0.80 (0.68-0.88), 0.83 (0.73-0.90), 19.06 (7.87-46.19) and 0.88 (0.85–0.91), respectively. There was significant heterogeneity among the included studies but no threshold effect in the meta-analysis. The result of subgroup analysis demonstrated that radiomics based on unenhanced and contrast-enhanced CT possessed higher diagnostic performance, and second-order or higher-order features could enhance the diagnostic sensitivity but also increase the false positive rate. No significant difference in diagnostic ability was observed between studies with machine learning and those without.

**Conclusions:**

The methodological quality and risk of bias of studies investigating the diagnostic performance of radiomics in adrenal tumors should be further improved in the future. CT-based radiomics has the potential benefits in differentiating malignant from benign adrenal tumors. The heterogeneity between the included studies was a major limitation to obtaining more accurate conclusions.

**Systematic Review Registration:**

https://www.crd.york.ac.uk/PROSPERO/ CRD 42022331999 .

## Introduction

Due to the increasing use of abdominal imaging, the discovery of adrenal incidentalomas has kept rising. It is reported that adrenal incidentalomas account for 4-5% of patients without malignancy (1). Although most adrenal masses are benign and non-functional, their functional status and malignant potential should be evaluated when they are detected, according to the latest recommendations (2). However, it is challenging for radiologists to accurately diagnose adrenal masses *via* conventional imaging assessments (3, 4). To begin, imaging features of pitfalls and mimics that are related to various abnormalities and aberrant appearances may potentially lead to misdiagnosis (3). For example, large adenomas usually present as heterogeneous masses on computed tomography (CT) images can not be easily differentiated from adrenocortical carcinoma visually (5, 6). Secondly, conventional imaging assessments depend largely on the experience and knowledge level of the radiologist. Consequently, exploring better approaches to improve the diagnostic value of adrenal imaging is crucial, considering that inappropriate diagnosis can lead to increased treatment costs or unnecessary examination (7).

Radiomics, first pioneered by Philippe Lambin, generally aims to extract quantitative and reproducible data that are imperceptible to the human eye from biomedical images for a series of medical purposes (8, 9). Extracted features, divided into shape-based, first-, second-, and higher-order statistics, can be translated into high-throughput and quantitative data for analysis (10, 11). The features that contribute the most to the objective will be selected for constructing the model *via* statistical approaches and artificial intelligence. Furthermore, radiomics features may achieve complementarity and improve accuracy when combined with clinically acquired, treatment-related, and genomic data (12). As artificial intelligence advances by leaps and bounds, radiomics has been extensively tested and applied in various aspects of oncology, including diagnosis, classification, and prognosis prediction (10). Recently, an increasing number of studies also established that radiomics could offer a risk-free and efficient method to increase the value of diagnostic imaging of adrenal masses. Nakajo et al. investigated the diagnostic performance of standardized uptake value (SUV)-related and texture parameters of F-18-fluorodeoxyglucose positron emission tomography/computedtomography (FDG PET/CT) between benign and metastatic adrenal tumors (13). In one study, texture analysis was applied to evaluate CT-abnormal adrenal glands in order to differentiate between malignant and benign tumors in patients with lung cancer (14). Moreover, Kong et al. designed a radiomic-based nomogram for pheochromocytoma diagnosis and achieved robust performance (15).

Although radiomics offers a relatively objective and quantitative diagnostic pattern, it is also subjected to data collection, radiomics characteristics processing, and modeling methods. Considering that the quality and results of published studies are mixed, diagnostic performance and feasibility of radiomics in adrenal masses remain elusive. Hence, the aim of the present review was to assess the methodological quality and risk of bias of radiomics studies investigating diagnostic performance in adrenal masses and to determine the potential diagnostic value of radiomics in adrenal tumors by quantitative analysis.

## Materials and methods

This review followed the Cochrane Handbook for Systematic Reviews of Interventions and was conducted in accordance with the PRISMA-DTA (Preferred Reporting Items for Systematic Reviews and Meta-analysis for Diagnostic Test Accuracy) statement (16, 17). The protocol of this review is available through PROSPERO (CRD 42022331999).

### Literature search

PubMed, Embase, Web of Science, and Cochrane Library databases were searched by two independent observers to identify eligible studies in May 2022. Additionally, the reference lists of the included studies were manually searched for studies that might meet the inclusion criteria.

### Study selection

The titles and abstracts of potentially relevant studies were screened by two reviewers (HZ and HL) independently. Then, the same two reviewers analyzed the full texts of eligible studies and determined the pieces of literature that met the inclusion criteria. Discrepancies between the two investigators were resolved by consensus with a third reviewer (JP).

All single, comparative studies, and primary studies that met the following PICO criteria were selected:

P (patients): Patients with benign or malign adrenal tumors;
I (interventions): Radiomics or texture analysis;
C (comparison): Standard-of-care imaging including computed tomography (CT) and magnetic resonance imaging (MRI), and positron emission tomography/computedtomography (PET/CT);
O (outcome): Histologic typing (including differentiation between different adrenal masses and differentiation between benign and malign adrenal tumors).

The exclusion criteria were as follows: (a) letters, reviews, editorials, expert opinions, case reports, meeting abstracts and comments; (b) non-human research; (c) the study was not written in English. The full search terms are outlined in **Table S1**.

### Quality assessment

The Radiomics Quality Score (RQS) and Quality Assessment of Diagnostic Accuracy Studies 2 (QUADAS-2) tools were utilized to assess the methodological quality and risk of bias of the included studies, respectively (18, 19). RQS comprises a total of 16 criteria, and the score of each item corresponds to the importance of the methodological quality of the study. The total score ranges from -8 to +36 points, with -8 to 0 points defined as 0% and 36 as 100% (16). The QUADAS-2 tool includes four evaluation criteria: (a) patient selection; (b) index test; (c) reference standard; and (d) flow and timing. Two independent reviewers (HZ and HL) performed the quality assessment, and disagreements between the two reviewers were resolved by consensus with a third reviewer (JP).

### Meta-analysis

A meta-analysis may be performed only when a sufficient number of studies attempt to answer a similar question. In this study, we performed a meta-analysis of all studies investigating the diagnostic performance of CT-based radiomics between malign and benign adrenal tumors. Data from all the eligible studies were extracted by two independent reviewers (HZ and HL). Discrepancies were resolved by consensus with a third reviewer (JP). Only studies from which a two-by-two contingency table could be extracted or reconstructed were included. If there were multiple models in the study, only the one with the highest area under the curve (AUC) was extracted. Moreover, the data from the model with the highest Youden’s Index was selected if AUC was not reported. When multiple publications were from the same research, only the study with the higher methodological quality was included.

### Statistical analysis

Pooled sensitivity, specificity, diagnostic odds ratio (DOR), positive likelihood ratio (PLR), and negative likelihood ratio (NLR) with 95% confidence intervals (CIs) were employed to quantify the diagnostic performance. In addition, diagnostic accuracy was outlined by the summary receiver operating characteristic curve (SROC) and area under the curve (AUC). The heterogeneity of studies was assessed by calculating the *I* ^2^ index, where an *I* ^2^ value of 0–25% represents insignificant heterogeneity, >25–50% indicates low heterogeneity, >50–75% indicates moderate heterogeneity, and >75% indicates high heterogeneity (20). A *p* < 0.05 was considered statistically significant. A random-effects model was employed to evaluate effect size and pool studies. Forest plots were constructed for visualization of the results. Spearman’s correlation coefficient was used to assess the threshold effect between sensitivity logit and (1-specificity) logit. In order to investigate the source of heterogeneity, a subgroup analysis was also conducted with the following covariates: (a) CT Type; (b) CT Feature Type; (c) Machine Learning; (d) Reference. The sensitivity analysis was performed by eliminating the included studies one after another. Publication bias was explored using the Deeks’ funnel plot, and statistical significance was assessed by Deeks’ asymmetry test. Clinical utility was examined using a Fagan plot, which provided the posttest probability when pretest probabilities were calculated (21).

Stata software (Stata Corporation, College Station, TX, USA, version 16.0) and the Open Meta-analyst (a completely open-source, cross-platform software) were used to conduct the meta-analysis. The interclass correlation coefficient (ICC), which described inter-rater agreement for the RQS and QUADAS-2 and Spearman’s correlation coefficient, was determined by SPSS software (IBM, Armonk, NY, USA, version 25.0).

## Results

### Included studies

**Figure 1:**
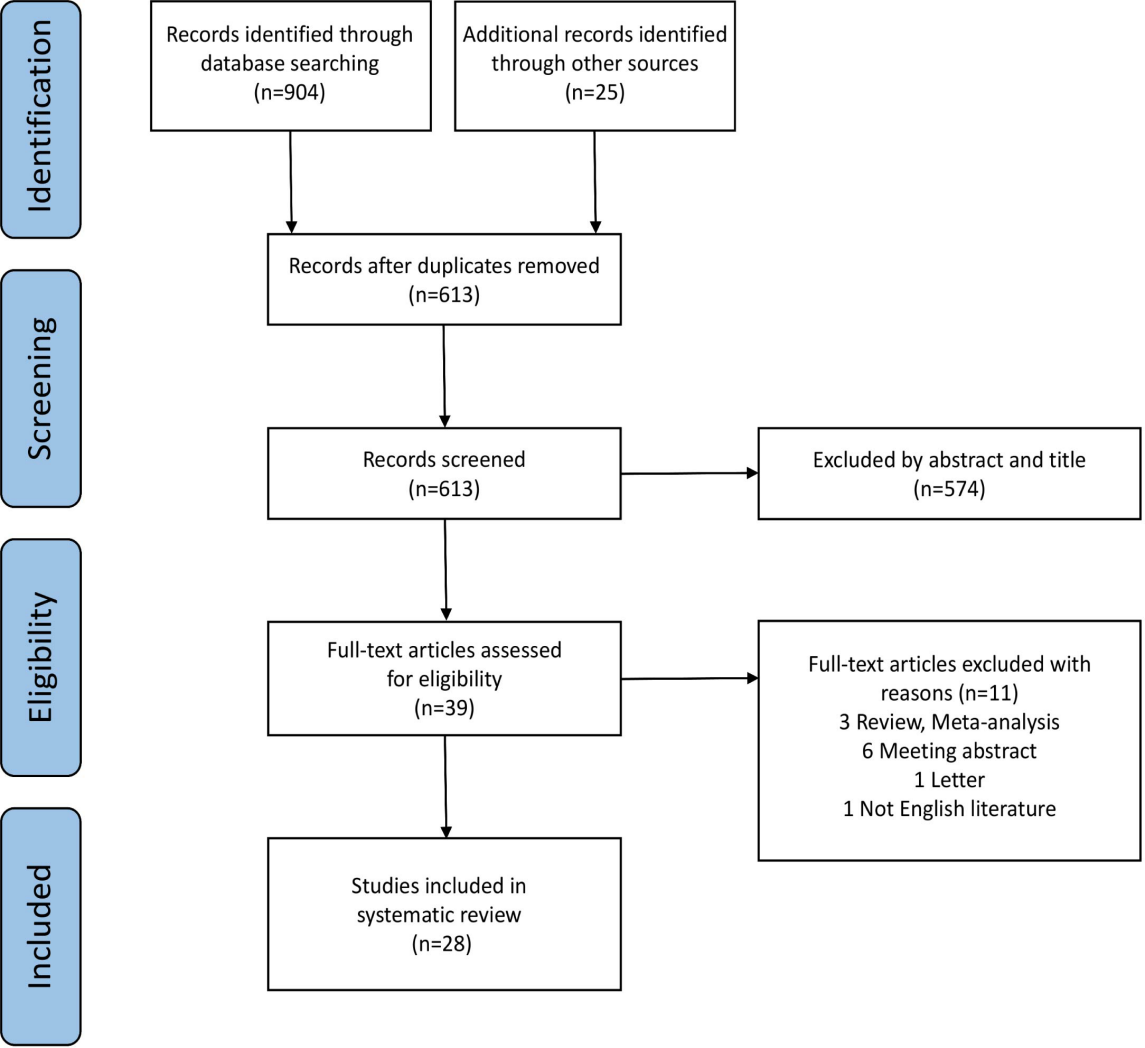
Flow diagram of study selection.

The PRISMA flow-chart of the literature search of this systematic review and meta-analysis is presented in **Figure 1**. 613 studies were screened following the removal of 316 duplicate records. Then, 574 articles were excluded by evaluating the abstract and title. After thoroughly screening the full-text, 3 studies were excluded for being reviews or meta-analyses; 6 for being meeting abstracts; one for being a letter; and one for being in a non-English language. Eventually, 28 studies were enrolled in this research. **Table 1** summarizes the characteristics of the included studies.

**Table 1:** Characteristics of the included studies.

Study ID	Ref	Study Design	Diagnostic Subject	Sample Size	Image Modality	Segmentation Method (Software/Algorithm)	Feature Extraction	Features Type	Modeling method	Reference Standard	Validation
Andersen et al. (2021)	(14)	Retrospective	Adrenal metastases/Benign lesions	160	Contrast-enhanced CT	Semi-automatic (Philips Intellispace Tumor Tracking)	TexRAD	First-order and higher-order	Binary logistic regression	Histopathology	NR
Chai et al. (2017)	(22)	Retrospective	Aldosterone-producing adenomas/Pheochromocytomas/Cushing adenomas	218	Unenhanced and contrast-enhanced CT	Automatic (Multiscale sparse representations)	NR	Shape-based, first-order and second-order	Support vector machine, radial basis function network (ML)	Histopathology	Internal validation
Elmohr et al. (2019)	(23)	Retrospective	Adrenocortical carcinomas/Adrenocortical adenomas	54	Unenhanced and contrast- enhanced CT	Manual (Amira Software)	PyRadiomics	Shape-based, first-order and second-order	Logistic regression, boruta random forest	Histopathology	Internal validation
Ho et al. (2019)	(24)	Retrospective	Adrenal malignancy/Lipid-poor adenoma	23	Unenhanced and contrast-enhanced CT, MRI 3T or 1,5T T1 in- and opposed-phase	Manual (Seg3D)	Lesion Tool (software developed by the authors)	Shape-based, first-order and second-order	Logistic regression	Histopathology	NR
Kong et al. (2022)	(15)	Retrospective	Pheochromocytoma/Other adrenal lesions	309	MRI 3T T2w	Semi-automatic (3D Slicer)	3D Slicer	Shape-based, first-order, second-order and higher-order	Logistic regression	Histopathology	Internal and external validation
Koyuncu et al. (2019)	(25)	Retrospective	Adrenal malignant/Benign lesions	114	Contrast-enhanced CT	Semi-automatic (AbSeg)	MATLAB	Shape-based, first-order, second-order and higher-order	Bounded particle swarm optimisation-neural network	NR	Internal validation
Li et al. (2018)	(26)	Retrospective	Adrenal malignant/Benign lesions	210	Unenhanced and contrast-enhanced CT	Manual (NR)	NR	Second-order	Bayesian spatial gaussian process classifier	Histopathology	NR
Liu et al. (2021)	(27)	Retrospective	Adrenal Adenoma/Pheochromocytoma	60	MRI 3T T1 in- and opposed-phase, T2w	Manual (Mazda)	MaZda	First-order	Support vector machine (ML)	Histopathology	Internal validation
Nakajo et al. (2017)	(13)	Retrospective	Adrenal metastases/Benign lesions	35	FDG PET/CT	Semi-automatic (Advantage Windows Workstation)	Python	First-order	NR	Clinical and imaging follow-ups	NR
Moawad et al. (2021)	(28)	Retrospective	Adrenal malignant/Benign lesions	40	Unenhanced and contrast-enhanced CT	Manual (Amira Software)	PyRadiomics	Shape-based, first-order, second-order and higher-order	Random forest (ML)	Histopathology	Internal validation
Rocha et al. (2018)	(29)	Retrospective	Adrenal adenomas/Malignant lesions	108	Unenhanced CT	Manual (OsiriX Software)	OsiriX	First-order	NR	Histopathology or follow-up imaging	NR
Romeo et al. (2018)	(30)	Retrospective	Lipid-rich/Lipid-poor/Nonadenoma adrenal lesions	60	MRI 3T T1w, T2w	Manual (ITK-SNAP)	3D Slicer	First-order and second-order	J48 classifier, Weka software (ML)	Histopathology	Internal validation
Schieda et al. (2017)	(31)	Retrospective	Adrenal metastases/Adrenal adenoma	44	MRI 1.5T or 3T T1 in- and opposed-phase, T2w, GRE	Manual (Image J)	Image J	First-order	Logistic regression	Histopathology or follow-up imaging	NR
Shi et al. (2019)	(32)	Retrospective	Adrenal metastases/Benign lesions	265	Unenhanced and contrast-enhanced CT	Manual (NR)	TexRAD	First-order and higher-order	Logistic regression, support vector machin (ML)	Histopathology or follow-up imaging	Internal validation
Shoemaker et al. (2018)	(33)	Retrospective	Adrenal malignant/Benign lesions; Adrenal functioning/Non-functioning lesions	377	Unenhanced CT	NR	NR	First-order and second-order	Logistic regression	Histopathology	Internal validation
Stanzione et al. (2021)	(34)	Retrospective	Adrenal malignant/Benign lesions	55	MRI 3T T1w, T2w	Manual (ITK-SNAP)	PyRadiomics	Shape-based, first-order, second-order and higher-order	Extra trees classifier (ML)	Histopathology or follow-up imaging	Internal validation
Szász et al. (2020)	(35)	Retrospective	Adrenal adenomas/Non-adenomas	233	Unenhanced CT	Manual (Advantage Windows workstation)	“Volume Histogram” tool	First-order	NR	Histopathology	NR
Torresan et al. (2021)	(36)	Retrospective	Adrenocortical carcinomas/Adenoma	19	Unenhanced and contrast-enhanced CT	Manual (PMOD)	PMOD	First-order and second-order	K-means clustering technique (ML)	Histopathology or follow-up imaging	NR
Tu et al. (2018)	(37)	Retrospective	Adrenal metastases/Adenomas	76	Contrast-enhanced CT	Manual (ImageJ)	Image J	First-order	Logistic regression	Previously described imaging thresholds or follow-up imaging	NR
Tu et al. (2020)	(38)	Retrospective	Adrenal Metastases/Lipid-poor adenomas	63	MRI 1.5T or 3T T1w, T2w, GRE	Manual (Image J)	Image J	First-order	Logistic regression	Histopathology or follow-up imaging	Internal validation
Tüdös et al. (2019)	(39)	Retrospective	Adrenal lipid-poor adenomas/Non-adenomas	163	Unenhanced CT	Manual (Advantage Windows workstation)	“Volume Histogram” tool	First-order	NR	Histopathology or follow-up imaging	NR
Umanodan et al. (2017)	(40)	Retrospective	Pheochromocytomas/Adrenal adenomas	52	MRI 3T ADC	Manual (Synapse Vincent software)	Synapse Vincent software	First-order	NR	Histopathology or follow-up imaging	NR
Wu et al. (2020)	(41)	Retrospective	Adrenal adenoma/Nonadenoma	94	Unenhanced CT	Manual (PACS software)	PACS software	First-order	NR	Histopathology or follow-up imaging	NR
Yi et al. (2018)	(42)	Retrospective	Pheochromocytomas/Adrenal lipid-poor adenomas	110	Unenhanced CT	Manual (MaZda)	MaZda	First-order, second-order and higher-order	Logistic regression (ML)	Histopathology	NR
Yi et al. (2018) (2)	(43)	Retrospective	Pheochromocytoma/Adrenal lipid-poor adenoma	265	Unenhanced and contrast-enhanced CT	Manual (MaZda)	MaZda	First-order, second-order and higher-order	Lasso, logistic regression	Histopathology	Internal validation
Yu et al. (2020)	(44)	Retrospective	Adrenal malignant/Benign lesions	125	Contrast-enhanced CT	Manual (TexRAD)	TexRAD	First-order and higher-order	NR	Histopathology or follow-up imaging	NR
Zhang et al. (2017)	(45)	Retrospective	Pheochromocytomas/Lipid-poor adrenocortical adenoma	164	Unenhanced and contrast-enhanced CT	Manual (TexRAD)	TexRAD	First-order and higher-order	NR	Histopathology	NR
Zheng et al. (2020)	(46)	Retrospective	Aldosterone-producing/Cortisol-producing functional adrenocortical adenomas	83	Unenhanced and contrast-enhanced CT	Manual (ITK-SNAP)	NR	Shape-based, first-order	Lasso, logistic regression (ML)	Histopathology	Internal validation

Ref, reference; NR, not report; ML, machine learning; PACS, picture archiving and communication system.

All 28 studies were retrospective cohort studies, and the sample size (number of lesions) ranged from 19 to 377. Most objectives of the included studies were differentiation between benign and malignant adrenal neoplasms by radiomics, followed by the differentiation between pheochromocytoma and adenoma. Other studies distinguished adrenal adenomas from non-adenomas or identified subtypes of adrenal adenomas. The majority of studies focused on the diagnostic performance of radiomics using CT imaging (n=19), while a quarter was based on magnetic resonance imaging (n=7). Additionally, one study explored the use of radiomics based on a combination of CT and MR and one based on FDG PET/CT. More than 78% (22/28) of studies used manual segmentation. Radiomics feature types used by different studies varied. Interestingly, over half of the included studies (n=17) extracted second or higher-order features for analyses. As for the modeling method, 12 studies conducted logistic regression, eight studies did not provide relevant information and the remainder employed other algorithms such as support vector machine, random forest, extra trees classifier and so forth. More than half studies utilized histopathology as the gold reference (n=15). Ten studies combined histopathology and follow-up imaging. Two articles exclusively considered clinical and imaging follow-ups, and one study failed to report the reference standard. Outcomes of the included studies are summarized in **Table 2**.

**Table 2:** Outcomes of the included studies.

Study ID	P	N	TP	FP	TN	FN	Sensitivity, %	Specificity, %	Accuracy, %	AUC	95%CI
Andersen et al. (2021)	71	89	41	20	69	30	58	77	68	0.730	–
Chai et al. (2017)	–	–	–	–	–	–	–	–	81.8 ~ 95.4	–	–
Elmohr et al. (2019)	–	–	–	–	–	–	81	83	82	0.890	–
Ho et al. (2019)	8	15	–	–	–	–	–	–	80	–	–
Kong et al. (2022)	–	–	–	–	–	–	85.7	75	84	0.906	0.841-0.971
Koyuncu et al. (2019)	12	45	9	8	37	3	75	82.2	80.7	0.786	–
Li et al. (2018)	96	114	91	37	77	5	94.8	67.5	80	–	–
Liu et al. (2021)	–	–	–	–	–	–	–	–	85	0.917	–
Nakajo et al. (2017)	22	13	22	2	11	0	100	84.6	94.3	0.970	0.840-0.990
Moawad et al. (2021)	19	21	16	6	15	3	84.2	71.4	77.5	0.850	–
Rocha et al. (2018)	88	20	77	1	19	11	87.5	95	88.9	–	–
Romeo et al. (2018)	–	–	–	–	–	–	–	–	80	–	–
Schieda et al. (2017)	15	29	14	4	25	1	93.3	86.2	88.6	0.970	0.930-1.000
Shi et al. (2019)	101	164	78	37	127	23	77	77	77.4	0.850	0.800-0.890
Shoemaker et al. (2018)	–	–	–	–	–	–	–	–	–	0.780~1.000	–
Stanzione et al. (2021)	18	37	–	–	–	–	–	–	0.91	0.970	0.870-1.000
Szász et al. (2020)	123	110	–	–	–	–	–	–	–	0.919	–
Torresan et al. (2021)	8	10	7	1	9	1	87.5	90	88.9	–	–
Tu et al. (2018)	40	36	19	9	27	21	47.5	75	60.5	0.650	0.520-0.770
Tu et al. (2020)	40	23	30	0	23	10	75	100	84.1	0.950	0.910-0.990
Tüdös et al. (2019)	83	80	44	1	79	39	53	98.8	75.5	–	–
Umanodan et al. (2017)	39	13	37	1	12	2	94.9	92.3	94.2	0.920	–
Wu et al. (2020)	58	36	51	16	20	7	87.9	55.6	75.5	0.740	–
Yi et al. (2018)	29	79	25	2	77	4	86.2	97.5	94.4	0.952	0.897-1.000
Yi et al. (2018) (2)	67	145	64	14	131	3	95.5	90.3	92	0.957	–
Yu et al. (2020)	81	44	66	0	44	15	81	100	88	0.970	0.940-0.990
Zhang et al. (2017)	98	66	78	11	55	20	79.6	83.3	81.1	0.860	0.810-0.910
Zheng et al. (2020)	–	–	–	–	–	–	91.5	92.8	92.2	0.902	0.822-0.982

P,condition positive; N, condition negative; TP, true positive; FP, false positive; TN, true negative; FN, false negative; AUC, area under the receiver operating characteristic; CI, confidence interval.

### Data quality assessment

The included studies achieved an mean ± standard deviation RQS of 5.11 ± 7.70, a median of 3.5, interquartile range 14, and a range of -5 to 25. The mean RQS proportion was 14.2%, with a maximum of 69.4%. The mode scores for the 16 dimensions are summarized in **Table 3**. The individual scores of each study and final scores of RQS are presented in **Tables S2 and S3**, respectively.

**Table 3:** Elements of the RQS and average rating achieved by the studies included in this systematic review.

RQS scoring item	Interpretation	Mode
Image Protocol	+1 for well documented protocols, +1 for publicly available protocols	1
Multiple Segmentations	+1 if segmented multiple times (different physicians, algorithms, or perturbation of regions of interest)	1
Phantom Study	+1 if texture phantoms were used for feature robustness assessment	0
Multiple Time Points	+1 multiple time points for feature robustness assessment	0
Feature Reduction	−3 if nothing, +3 if either feature reduction or correction for multiple testing	3
Non Radiomics	+1 if multivariable analysis with non-radiomics features	0
Biological Correlates	+1 if present	0
Cut-off	+1 if cutoff either pre-defined or at median or continuous risk variable reported	0
Discrimination and Resampling	+1 for discrimination statistic and statistical significance, +1 if resampling applied	1
Calibration	+1 for calibration statistic and statistical significance, +1 if resampling applied	0
Prospective	+7 for prospective validation within a registered study	0
Validation	−5 if no validation/+2 for internal validation/+3 for external validation/+4 two external validation datasets or validation of previously published signature/+5 validation on ≥3 datasets from >1 institute	-5
Gold Standard	+2 for comparison to gold standard	2
Clinical Utility	+2 for reporting potential clinical utility	2
Cost-effectiveness	+1 for cost-effectiveness analysis	0
Open Science	+1 for open-source scans, +1 for open-source segmentations, +1 for open-source code, +1 open-source representative segmentations and features	0

**Figure 2:**
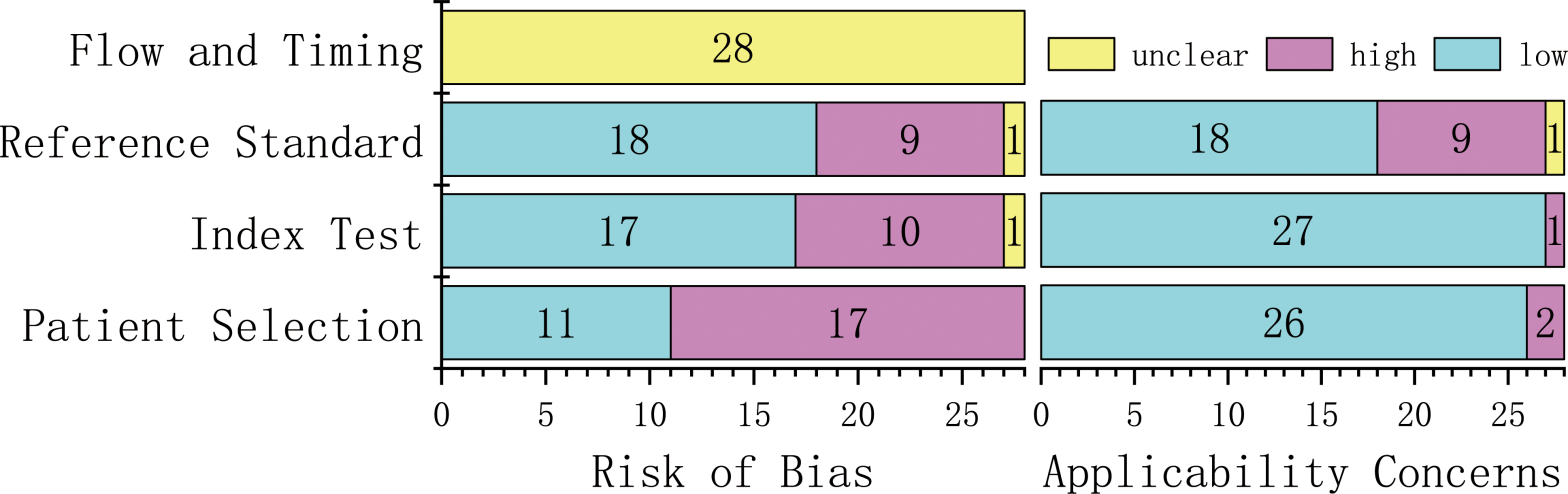
The risk of bias and concerns regarding applicability of 28 included studies.

The majority of studies provided details about the imaging scheme, applied discrimination statistics and achieved their potential clinical utility. Conversely, none of the included studies employed phantoms, considered biological correlates or assessed the repeatability of radiomics analysis at multiple time points. Moreover, feature reduction or adjustment of multiple tests were performed in 16/28 (57%) studies, and non-radiomics features were applied in 3/28 (11%) studies. Only a few studies conducted model calibration, assessed the cost-effectiveness and publicly shared segmentations or code. The inter-reader agreement was found to be moderate to excellent for radiomics features in 39% (11/28) of the included studies. Nevertheless, validation of more than half of the included studies was missing (15/28, 50%). Only one study (28) compared the diagnostic performance of the classifier with an expert radiologist, but no significant differences were noted. In general, the quality of included articles was acceptable, and the assessment of the risk of bias and applicability of the 28 included studies are illustrated in **Figure 2**. The detail of the individual and final evaluation of the risk of bias and applicability concerns are presented in **Tables S4 and S5**, respectively.

Inter-rater agreements of RQS and QUADAS-2 were also assessed by the ICC. The ICC for the RQS was 0.94 (95% CI 0.93–0.95). Six criteria of RQS reached a moderate agreement, while ten items achieved substantial or almost perfect agreement (**Table 4**). The ICC for the QUADAS-2 was 0.96 (95% CI 0.95–0.97). Except for two dimensions reaching a moderate agreement, the others exceeded 75% agreement (**Table 5**).

**Table 4:** Inter-rater agreement in RQS assessment.

RQS scoring item	ICC (95% CI)
Image Protocol	0.52 (0.19-0.75)
Multiple Segmentations	0.93 (0.86-0.97)
Phantom Study	1.00 (1.00–1.00)
Multiple Time Points	1.00 (1.00–1.00)
Feature Reduction	0.86 (0.72-0.93)
Non Radiomics	0.79 (0.59-0.90)
Biological Correlates	1.00 (1.00–1.00)
Cut-off	0.63 (0.34-0.81)
Discrimination and Resampling	0.52 (0.19-0.75)
Calibration	1.00 (1.00–1.00)
Prospective	1.00 (1.00–1.00)
Validation	1.00 (1.00–1.00)
Gold Standard	0.54 (0.22–0.76)
Clinical Utility	0.61 (0.32-0.80)
Cost-effectiveness	1.00 (1.00–1.00)
Open Science	0.79 (0.59-0.90)

CI: confidence interval, RQS: Radiomics Quality Score.

**Table 5:** Inter-rater agreement in QUADAS-2 assessment.

RQS scoring item	ICC (95% CI)
Risk of Bias - Patient Selection	0.79 (0.60-0.90)
Risk of Bias - Index Test	0.94 (0.87-0.97)
Risk of Bias - Reference Standard	1.00 (1.00–1.00)
Risk of Bias - Flow and Timing	1.00 (1.00–1.00)
Applicability Concerns- Patient Selection	0.52 (0.19-0.75)
Applicability Concerns- Index Test	0.66 (0.39-0.83)
Applicability Concerns- Reference Standard	1.00 (1.00–1.00)

CI: confidence interval, QUADAS-2: Quality Assessment of Diagnostic Accuracy Studies-2.

### Meta-analysis

**Figure 3:**
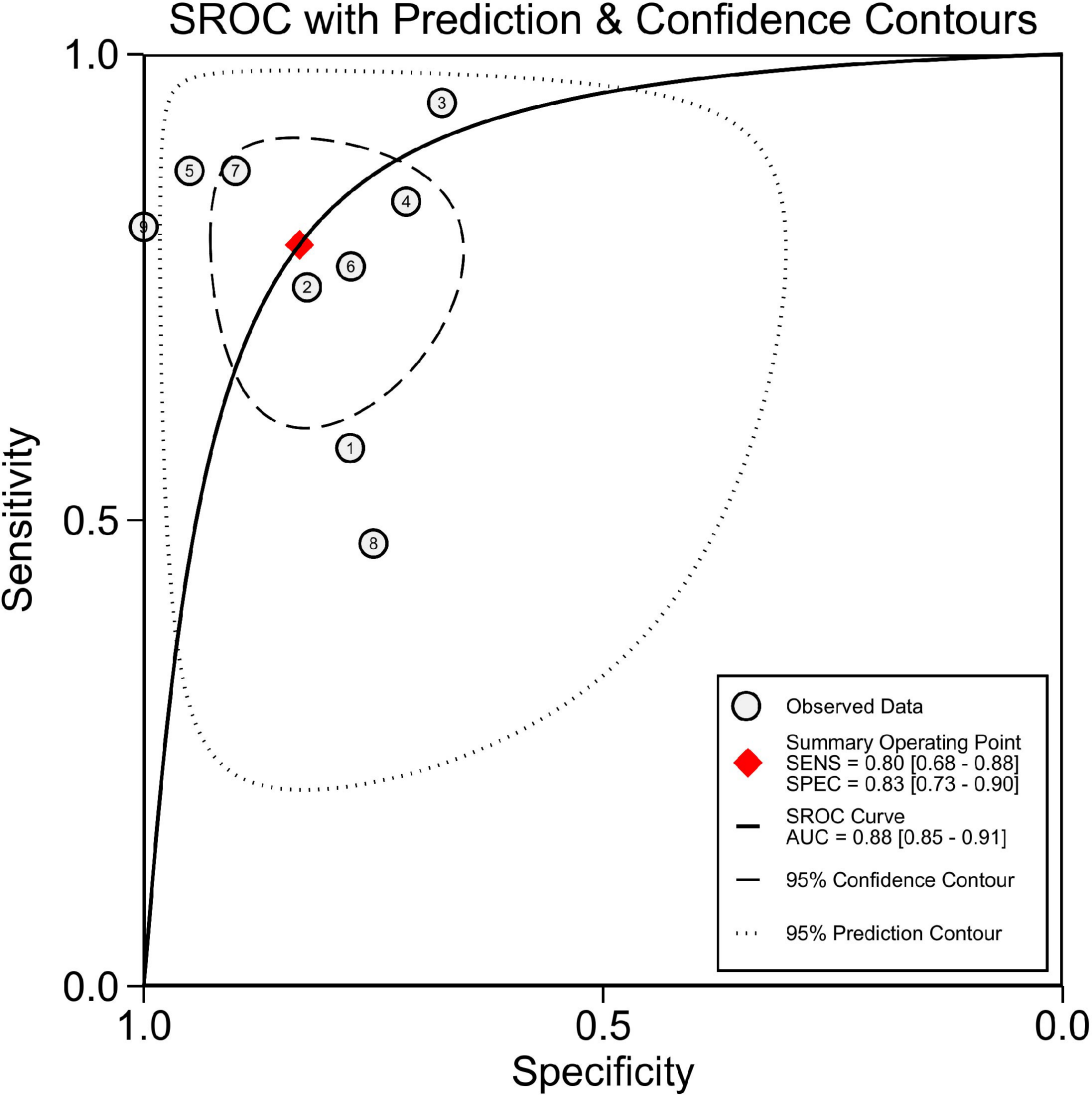
Summary receiver operating characteristic (SROC). AUC, area under the curve.

**Figure 4:**
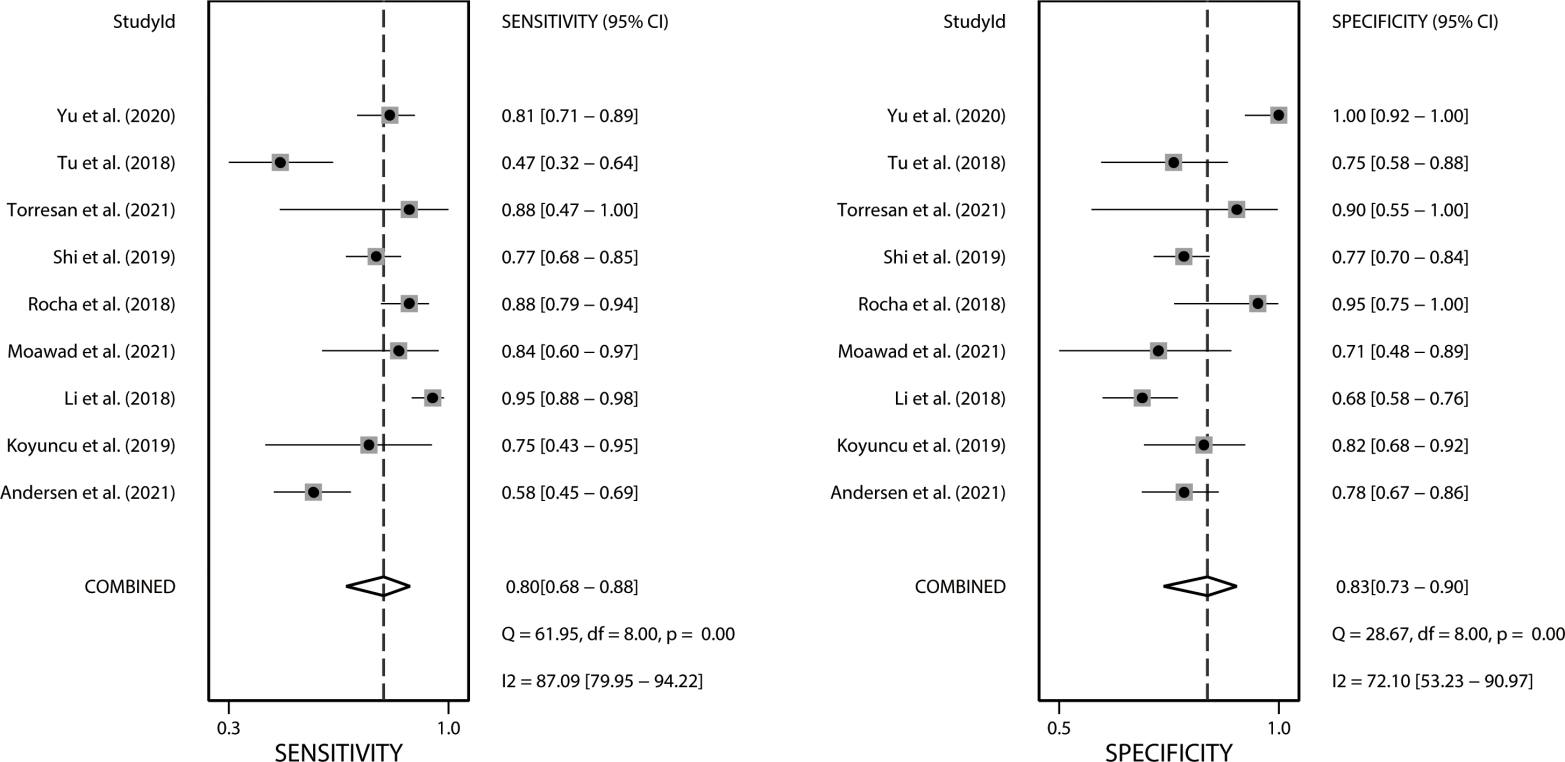
Forest plots of the sensitivity and specificity of CT-based radiomics in differentiating malignant from benign adrenal tumors. *I* ^2^ >50% indicates substantial heterogeneity among included studies.

We performed a meta-analysis investigating the use of CT-based radiomics in differentiating malignant from benign adrenal tumors and enrolled nine eligible studies, from which a two-by-two contingency table could be extracted or reconstructed. As shown in **Table 6**, the mean values and 95% CIs of the pooled sensitivity, specificity, PLR, NLR, and DOR for the radiomics signature based on CT in differentiating malignant adrenal tumors from benign tumors were 0.80 (0.68-0.88), 0.83 (0.73-0.90), 4.70 (2.80-8.00), 0.25 (0.15-0.41) and 19.06 (7.87-46.19) respectively. The summary receiver operating characteristic curve showed an overall pooled AUC of 0.88 (95% CI 0.85–0.91) (**Figure 3**). Significant heterogeneity in sensitivity (*I* ^2^ = 87.09%) and specificity (*I* ^2^ = 72.1%) were noted, as depicted in **Figure 4**. Consequently, diagnostic threshold analysis was carried out, which revealed that there was no threshold effect, given that the Spearman’s correlation coefficient was -0.036 and the *p*-value was 0.932. In order to further explore the cause of heterogeneity, subgroup analysis was also performed, as outlined in **Table 6**.

**Table 6:** The results of subgroup analysis.

Analysis	No. of study	Sensitivity	Specificity	PLR	NLR	DOR
**CT Type**						
Contrast-enhanced CT	4	0.66(0.47-0.80)	0.80(0.70-0.88)	3.15(1.69-5.89)	0.50(0.35-0.72)	9.02(2.59-31.43)
Unenhanced and contrast-enhanced CT	4	0.87(0.72-0.95)	0.74(0.66-0.80)	3.15(2.60-3.82)	0.17(0.07-0.41)	18.89(8.96-39.85)
Unenhanced CT	1	0.88(0.79-0.93)	0.95(0.72-0.99)	17.50(2.59-118.41)	0.13(0.02-0.89)	133.00(16.16-1094.59)
**CT Feature Type**						
With second-order or higher-order features	7	0.81(0.69-0.89)	0.77(0.70-0.83)	3.21(2.55-4.04)	0.23(0.11-0.47)	16.97(7.56-38.12)
Only first-order	2	0.72(0.25-0.95)	0.86(0.51-0.97)	4.77(0.56-40.72)	0.39(0.08-1.86)	16.91(0.38-761.14)
**Machine Learing**						
Not use machine learing	6	0.78(0.60-0.89)	0.79(0.69-0.87)	3.20(2.12-4.82)	0.26(0.09-0.76)	18.80(5.37-65.75)
Use machine learing	3	0.79(0.71-0.85)	0.77(0.71-0.83)	3.41(2.59-4.50)	0.28(0.21-0.37)	12.54(7.28-21.59)
**Reference**						
Histopathology	3	0.83(0.46-0.97)	0.72(0.65-0.78)	2.83(2.28-3.52)	0.21(0.05-0.81)	12.93(3.08-54.26)
Histopathology or follow-up imaging	4	0.82(0.76-0.86)	0.91(0.72-0.98)	9.33(2.60-33.52)	0.28(0.21-0.38)	59.05(9.39-371.52)
Previously described imaging thresholds or follow-up imaging	1	0.48(0.33-0.63)	0.75(0.59-0.86)	1.90(0.99-3.65)	0.70(0.36-1.35)	2.71(1.02-7.21)
Overall	9	0.80(0.68-0.88)	0.83(0.73-0.90)	4.70(2.80-8.00)	0.25(0.15-0.41)	19.06(7.87-46.19)

PLR, positive likelihood ratio; NLR, negative likelihood ratio; DOR, diagnostic odds ratio; The 95% confidence intervals are shown in parentheses.

Four studies with radiomics based on unenhanced and contrast-enhanced CT demonstrated higher sensitivity (0.87 vs. 0.66) but lower specificity (0.74 vs. 0.80) than studies using only contrast-enhanced CT. Studies (n=2) that only included first-order features had lower sensitivity (0.72 vs. 0.81) but higher specificity (0.86 vs. 0.77) compared to those that combined with second-order or higher-order features. Interestingly, the studies (n=3) that applied machine learning gained equivalent sensitivity (0.79 vs. 0.78) as well as specificity (0.77 vs. 0.79) compared to those did not use (n=6). Four studies that considered histopathology or follow-up imaging as a reference had higher specificity (0.91 vs. 0.72) and equivalent sensitivity (0.82 vs. 0.83) than studies (n=3) using only histopathology. The corresponding forest plots for sensitivity and specificity are delineated in **Figures S1–S4**.

**Figure 5:**
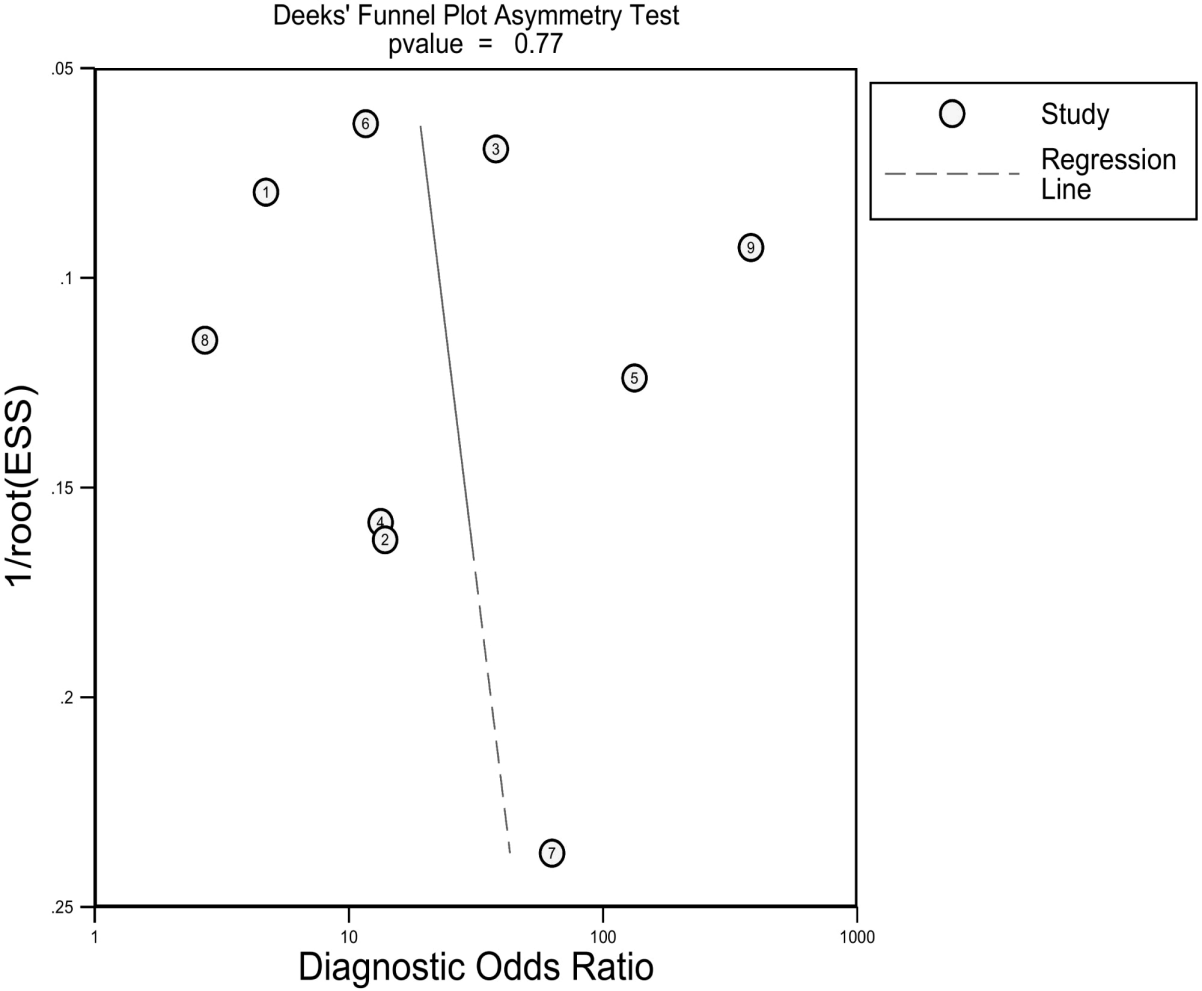
Deeks funnel plot reveals the possibility of publication bias is low with a p value of 0.77. ESS, effective sample size.

As shown in **Table S6**, we can hardly identify significant changes in the pooled effect value when eliminating studies one by one. There was no publication bias based on the Deeks’ funnel plot (*p*=0.77), as presented in **Figure 5**. Furthermore, the clinical utility was also evaluated using a Fagan plot. Using a CT-based radiomics model would increase the posttest probability to 54% from 20% with a PLR of 5 when the pretest was positive and reduce the posttest probability to 6% with an NLR of 0.25 when the pretest was negative, as depicted in **Figure S5**.

## Discussion

Radiomics has recently attracted the attention of oncology researchers, given that it can noninvasively and effectively reflect tumor heterogeneity, treatment response, prognosis, and other information (47–49). Published studies involving radiomics for adrenal tumors mainly focused on the differentiation of benign and malignant tumors and the differentiation of histological types of adrenal masses, but no clinical transformation or practical application has been described so far. Considering that the research of radiomics remains in its infancy, there are a host of problems to be addressed, such as the lack of a robust workflow based on standardized and strict methods to ensure the stability and reliability of the results (50, 51).

It is well-established that the quality of reporting of existing predictive models is poor (52). Lambin et al. proposed a comprehensive and clear standard to evaluate all aspects of predictive models in the field of radiomics to enhance their qualities (18). In our systematic review, the overall quality was relatively low (mean RQS of 5.11, ranging from -5 to 25). The primary causes impacting the RQS score included the absence of feature reduction, scarcity of open science and source, deficiency in internal or external validation and prospective data support, minimal consideration of cost-effectiveness, and so on. Reducing features that are poorly reproducible is crucial for reducing the risk of overfitting when the number of radiomics features exceeds the number of patients (53). 43% of studies did not conduct feature reduction or adjustment in our review owing to specific diagnostic algorithms or processing, which may partially undermine the stability of the models. Following internal and external validation, the diagnostic performance of the model can be confirmed. Furthermore, the practical application of radiomics in clinical practice also requires multi-center validation and prospective testing (54, 55). Regrettably, over half of the included studies failed to process validation attributable to the limited sample size. Most of the remaining studies merely conducted internal validation in a single center. Furthermore, only one study performed validation with multiple data sets and tested prospectively (15). Furthermore, comparing the diagnostic performance with the radiologist is also a pivotal step in verifying the performance of the model. Because only when the diagnostic effect is better than that of the radiologist can the superiority of radiomics be demonstrated. However, most studies did not compare the diagnostic performance with a radiologist. The choice of scanner manufacturer and model, 2D or 3D segmentation of the region of interest, acquisition, and reconstruction parameters all lead to the heterogeneity of imaging data. Most studies (25/28) provided image acquisition parameters in our review, but values varied considerably. Zwanenburg et al. designed the Image Biomarker Standardization Initiative (IBSI) to enhance the reproducibility of radiomics research, including establishing general feature naming, definition, general radiological image processing scheme, and so on (56). Thus, open science and the source of radiomics is the premise to realizing reproducibility. In the present review, only three included studies publicly shared segmentations or code. The challenge of open science and validation based on a sufficient sample size may hinder further development and practice of radiomics in the diagnosis of adrenal masses. Besides, the cost-effectiveness analyses of radiomics cannot be overlooked because it may boost the superiority of this technology.

Although radiomics studies differ methodologically from conventional trials, and there may be potential unsuitability of the QUADAS-2 tool, the results that reflected the risk of bias and applicability of included studies is advisable to some extent. The results of QUADAS-2 exposed that the risk of bias needs to be minimized in terms of patient selection, index test, and reference standards. The concerns regarding applicability are excellent except for the reference standard. The reliablity of individual ratings needs to be assessed by inter-rater agreement analysis. In this review, the ICC was applied to describe the inter-rater agreement of RQS and QUADAS-2. The fact that most items achieved substantial or almost perfect agreement while others had moderate agreement demonstrates that the scores accurately reflect the quality of the included studies.

Since there are few pieces of literature included in the meta-analysis, the results should be treated with caution. In our meta-analysis, radiomics technology showed promise for differentiating malignant from benign adrenal tumors, with a pooled sensitivity, specificity, and AUC of 0.8, 0.83, and 0.88, respectively. Nonetheless, it cannot be ignored that there was distinct heterogeneity between the studies. The threshold effect is one of the chief causes of heterogeneity in DTA studies (57). A threshold effect will result in a correlation coefficient between sensitivity and a false positive rate of 0.6 or higher (58). The result of the Spearman correlation coefficient showed no threshold effect in this meta-analysis. Consequently, we attempted to determine the causes of heterogeneity *via* subgroup analysis. Our results demonstrated that the radiomics group based on unenhanced and contrast-enhanced CT had a higher DOR than studies using contrast-enhanced CT only. This is likely due to the fact that unenhanced CT provides additional features for analysis and bring higher sensitivity. Different levels of radiomics features contain distinct dimensions of information regarding the lesion. First-order statistics features describe the distribution of voxel values without concern for their spatial relationships (11). Second-order statistics, which describe spatial relationships between voxels with similar gray levels within a lesion, can provide a measure of intralesional heterogeneity (11, 59). Higher-order statistics are obtained after imposing filter grids on an image, and the processing can confirm repetitive or non-repetitive patterns, suppress image noise, highlight details, and so on (60). According to our results, studies that only included first-order features had lower sensitivity but higher specificity compared to those that combined second-order or higher-order features. This finding signals that more complex and deeper texture features analyses can improve diagnostic sensitivity while also increasing the false positive rate. Since deeper texture features analyses inevitably yield a large number of unstable and unrepeatable features, advanced features have higher requirements for feature selection and modeling algorithms. Machine learning is a broad term for a class of statistical analysis algorithms that can iteratively improve the predictive performance of a model by “learning” from data (61). Reliable machine-learning approaches can drive the success of radiomic applications in clinical care (62). In our subgroup analysis, studies with machine learning achieved equivalent diagnostic performance to those without. However, the number of studies (n=3) is insufficient to represent the true impact of machine learning. We recommend that further studies be conducted to determine whether machine learning is beneficial to the diagnosis of adrenal tumors using radiomics. Some studies included in this meta-analysis enrolled patients without histopathology results and regarded follow-up imaging as the diagnostic reference (29, 32, 36, 37, 44). On the one hand, these studies may lower selection bias, as potential bias will be generated if studies only include patients who underwent surgeries (those with high suspicion of malignancy are more likely to be operated on). On the other hand, the diagnostic accuracy of this method based on follow-up images remains to be determined. The 2017 American College of Radiology white paper (63) suggests that stability for 1 year or more indicates that uncertain adrenal nodules are benign, whereas enlarged nodules are suspected to be malignant. However, benign tumors can also grow, and the threshold growth rate to consider malignancy remains unknown. Studies that regarded histopathology or follow-up imaging as the reference had higher diagnostic specificity than studies using histopathology only. The reason may be that the true negative ratio was overestimated since some follow-up imaging failed to detect potential malignancies. Additionally, the possibility that heterogeneity was caused by other factors that have not been considered cannot be ruled out.

To the best of our knowledge, there are two previous reviews related to similar topics. One study systematically reviewed the diagnostic accuracy of CT texture analysis in adrenal tumors (64). In another review, Stanzione et al. summarized the application of radiomics in adrenal cross-sectional imaging and assessed the methodological quality by RQS (65). Generally, more comprehensive and in-depth analyses of diagnostic performance of radiomics in adrenal masses were done in our study. First of all, we focused on diagnostic performance of radiomics in various radiological imaging of adrenal tumors. Secondly, RQS and QUADAS-2 of the included studies were independently evaluated by two reviewers. Besides, inter-rater agreement for RQS and QUADAS-2 were also assessed, which can reflect the true quality of the included studies better. In addition to a systematic review of the included studies, we also conducted a meta-analysis investigating the role of CT-based radiomics in differentiating malignant from benign adrenal tumors. Although the heterogeneity was significant, it reflected the diagnostic value of radiomics in differentiating benign and malignant adrenal masses to some extent.

There are several limitations of this review that warrant consideration. To begin, grey literature was not included in this review since it was limited to special circulation channels, which might have led to publication bias. Secondly, the overall quality of the included studies was not optimal (mean RQS 14.2%), which may have partly influenced the quality of the subsequent analysis. Thirdly, it is worthwhile mentioning the heterogeneity of studies included in the quantitative synthesis. Except for CT type, CT feature type, machine learning, and diagnostic reference, the heterogeneity may be pertinent to diversity in pathological types, methods of image segmentation and reconstruction, and feature extraction and modeling algorithms. However, because the subgroup distribution was scattered, we were unable to analyze these detailed features. Hence, the results of the quantitative analysis should be interpreted with caution. Fourthly, the diagnostic performance of radiomics between specific adrenal histologic types could not be assessed because of a lack of studies for the same objective. Lastly, given that only a few studies compared the diagnostic performance with a radiologist, the added value of radiomics in comparison to the accuracy of human assessment could not be explored.

## Conclusion

In conclusion, we systematically reviewed studies investigating the diagnostic performance of radiomics in adrenal masses and conducted a meta-analysis. Collectively, the results of quantitative synthesis outline the potential benefits of CT-based radiomics in differentiating malignant from benign adrenal tumors. However, the existing limitations of relevant studies, including the lack of validation and prospective tests, the lack of comparison with a radiologist, and the absence of a standardized radiomics process, hinder the further development of radiomics. We postulate that the translational gap between radiomics research and clinical applications in the field of adrenal tumors diagnosis will be overcome in the future by addressing the aforementioned shortcomings.

## Data availability statement

The original contributions presented in the study are included in the article/**Supplementary Material**, further inquiries can be directed to the corresponding author.

## Author contributions

Conceptualization and methodology: HZ and HL. Data analysis, drafting and revising: HZ, HL, and JP. Writing, editing, and revision of manuscript: HZ. Supervision, review, project administration and funding acquisition: JP. All authors contributed to the article and approved the submitted version.

## Funding

This work was supported by Sanming Project of Medicine in Shenzhen (00101100032).

## Conflict of interest

The authors declare that the research was conducted in the absence of any commercial or financial relationships that could be construed as a potential conflict of interest.

## Publisher’s note

All claims expressed in this article are solely those of the authors and do not necessarily represent those of their affiliated organizations, or those of the publisher, the editors and the reviewers. Any product that may be evaluated in this article, or claim that may be made by its manufacturer, is not guaranteed or endorsed by the publisher.
