# Supplementary material for: Diagnostic performance of radiomics in adrenal masses: A systematic review and meta-analysis
Source: Front Oncol. 2022 Sep 2;12:975183. doi: 10.3389/fonc.2022.975183 (PMC9478189; doi:10.3389/fonc.2022.975183)
Supplement: Supplementary file 1 [file DataSheet_2.docx]

| Table S1. Search terms | | | | |
| --- | --- | --- | --- | --- |
| Database | Hits | Search Date | Criterion | Search terms |
| Pubmed | 370 | 05/13/2022 |  |  |
|  |  |  | P | "Adrenal Gland Neoplasms"[Mesh] OR "Adrenal Cortex Neoplasms"[Mesh] OR "Adrenocortical Adenoma"[Mesh] OR "Adrenocortical Carcinoma"[Mesh] OR "Pheochromocytoma"[Mesh] OR Adrenal Gland Neoplasm*[tiab] OR Adrenal Neoplasm*[tiab] OR Adrenal Cancer*[tiab] OR "Cancer of the Adrenal Gland"[tiab] OR Adrenal Gland Cancer*[tiab] OR Adrenal Cortex Neoplasm*[tiab] OR Adrenocortical Cancer*[tiab] OR "Cancer of Adrenal Cortex"[tiab] OR "Cancer of the Adrenal Cortex"[tiab] OR Adrenal Cortex Cancer*[tiab] OR Adrenocortical Adenoma*[tiab] OR Adrenal Cortical Adenoma*[tiab] OR Conn Adenoma*[tiab] OR aldosteronoma*[tiab] OR Adrenocortical Carcinoma*[tiab] OR Adrenal Cortical Carcinoma*[tiab] OR Pheochromocytoma*[tiab] OR adrenal incidentaloma*[tiab] |
|  |  |  | I | "Image Processing, Computer-Assisted"[Mesh] OR Computer Assisted Image*[tiab] OR  Image Reconstruction*[tiab] OR  radiomics[tiab] OR textur*[tiab] OR |
|  |  |  |  | "Algorithms"[Mesh] OR Histogram*[tiab] OR Algorithm*[tiab] |
|  |  |  | C | "Tomography, X-Ray Computed"[Mesh] OR  "computed tomography"[tiab] OR  "Computerized Tomography"[tiab] OR "Computed X Ray Tomography"[tiab] OR  "Computerized X Ray Tomography"[tiab] OR  "Computer Assisted Tomography"[tiab] OR  "Computerized Axial Tomography"[tiab] OR  "Computer Axial Tomography"[tiab] OR "Computed Axial Tomography"[tiab] OR CT[tiab] OR  CAT[tiab] OR |
|  |  |  |  | "Magnetic Resonance Imaging"[Mesh] OR  "magnetic resonance"[tiab] OR  MRI[tiab] OR MR[tiab] |
| Embase | 250 | 05/13/2022 |  |  |
|  |  |  | P | "Adrenal Gland Neoplasm*" OR "Adrenal Neoplasm*" OR "Adrenal Cancer*" OR "Cancer of the Adrenal Gland" OR "Adrenal Gland Cancer*" OR "Adrenal Cortex Neoplasm*" OR "Adrenocortical Cancer*" OR "Cancer of Adrenal Cortex" OR "Cancer of the Adrenal Cortex" OR "Adrenal Cortex Cancer*" OR "Adrenocortical Adenoma*" OR "Adrenal Cortical Adenoma*" OR "Conn Adenoma*" OR "aldosteronoma*" OR "Adrenocortical Carcinoma*" OR "Adrenal Cortical Carcinoma*" OR "Pheochromocytoma*" OR "adrenal incidentaloma*" |
|  |  |  | I | "Computer Assisted Image*" OR "Image Reconstruction*" OR "radiomics" OR "textur*" OR "Histogram*" OR "Algorithm*" |
|  |  |  | C | "computed tomography" OR "Computerized Tomography" OR "Computed X Ray Tomography" OR "Computerized X Ray Tomography" OR "Computer Assisted Tomography" OR "Computerized Axial Tomography" OR "Computer Axial Tomography" OR "Computed Axial Tomography" OR "CT" OR  "CAT" OR "magnetic resonance" OR  "MRI" OR "MR" |
| Web of Science | 283 | 05/13/2022 |  |  |
|  |  |  | P | "Adrenal Gland Neoplasm*" OR "Adrenal Neoplasm*" OR "Adrenal Cancer*" OR "Cancer of the Adrenal Gland" OR "Adrenal Gland Cancer*" OR "Adrenal Cortex Neoplasm*" OR "Adrenocortical Cancer*" OR "Cancer of Adrenal Cortex" OR "Cancer of the Adrenal Cortex" OR "Adrenal Cortex Cancer*" OR "Adrenocortical Adenoma*" OR "Adrenal Cortical Adenoma*" OR "Conn Adenoma*" OR "aldosteronoma*" OR "Adrenocortical Carcinoma*" OR "Adrenal Cortical Carcinoma*" OR "Pheochromocytoma*" OR "adrenal incidentaloma*" |
|  |  |  | I | "Computer Assisted Image*" OR "Image Reconstruction*" OR "radiomics" OR "textur*" OR "Histogram*" OR "Algorithm*" |
|  |  |  | C | "computed tomography" OR "Computerized Tomography" OR "Computed X Ray Tomography" OR "Computerized X Ray Tomography" OR "Computer Assisted Tomography" OR "Computerized Axial Tomography" OR "Computer Axial Tomography" OR "Computed Axial Tomography" OR "CT" OR  "CAT" OR "magnetic resonance" OR "MRI" OR "MR" |
| Cochrane Library | 1 | 05/13/2022 |  |  |
|  |  |  | P | [mh "Adrenal Gland Neoplasms"] OR [mh "Adrenal Cortex Neoplasms"] OR [mh "Adrenocortical Adenoma"] OR [mh "Adrenocortical Carcinoma"] OR [mh "Pheochromocytoma"] OR ("Adrenal Gland Neoplasm*"):ti,ab,kw OR ("Adrenal Neoplasm*"):ti,ab,kw OR ("Adrenal Cancer*"):ti,ab,kw OR ("Cancer of the Adrenal Gland "):ti,ab,kw OR ("Adrenal Gland Cancer*"):ti,ab,kw OR ("Adrenal Cortex Neoplasm*"):ti,ab,kw OR ("Adrenocortical Cancer*"):ti,ab,kw OR ("Cancer of Adrenal Cortex"):ti,ab,kw OR ("Cancer of the Adrenal Cortex"):ti,ab,kw OR ("Adrenal Cortex Cancer*"):ti,ab,kw OR ("Adrenocortical Adenoma*"):ti,ab,kw OR ("Adrenal Cortical Adenoma*"):ti,ab,kw OR ("Conn Adenoma*"):ti,ab,kw OR ("aldosteronoma*"):ti,ab,kw OR ("Adrenocortical Carcinoma*"):ti,ab,kw OR ("Adrenal Cortical Carcinoma*"):ti,ab,kw OR ("Pheochromocytoma*"):ti,ab,kw OR ("adrenal incidentaloma*"):ti,ab,kw |
|  |  |  | I | [mh "Image Processing, Computer-Assisted"] OR ("Computer Assisted Image*"):ti,ab,kw OR ("Image Reconstruction*"):ti,ab,kw OR ("radiomics"):ti,ab,kw OR ("textur*"):ti,ab,kw OR [mh "Algorithms"] OR ("Histogram*"):ti,ab,kw OR ("Algorithm*"):ti,ab,kw |
|  |  |  | C | [mh "Tomography, X-Ray Computed"] OR ("computed tomography"):ti,ab,kw OR ("Computerized Tomography"):ti,ab,kw OR ("Computed X Ray Tomography"):ti,ab,kw OR ("Computerized X Ray Tomography"):ti,ab,kw OR ("Computer Assisted Tomography"):ti,ab,kw OR ("Computerized Axial Tomography"):ti,ab,kw OR ("Computer Axial Tomography"):ti,ab,kw OR ("Computed Axial Tomography"):ti,ab,kw OR ("CT"):ti,ab,kw OR ("CAT"):ti,ab,kw OR [mh "Magnetic Resonance Imaging"] OR ("magnetic resonance"):ti,ab,kw OR ("MRI"):ti,ab,kw OR ("MR"):ti,ab,kw |

| Table S2. Individual scores of RQS (HZ / HL) | | | | | | | | | | | | | | | | | | | | | | | | | | | | | | | | |
| --- | --- | --- | --- | --- | --- | --- | --- | --- | --- | --- | --- | --- | --- | --- | --- | --- | --- | --- | --- | --- | --- | --- | --- | --- | --- | --- | --- | --- | --- | --- | --- | --- |
| Study ID | *Image Protocol* | | *Multiple Segmentations* | | *Phantom Study* | | *Multiple Time Points* | | *Feature Reduction* | | *Non Radiomics* | | *Biological Correlates* | | *Cut-off* | | *Discrimination and Resampling* | | *Calibration* | | *Prospective* | | *Validation* | | *Gold Standard* | | *Clinical Utility* | | *Cost-effectiveness* | | *Open Science* | |
| Andersen et al. (2021) | 1 | 1 | 0 | 0 | 0 | 0 | 0 | 0 | 3 | 3 | 0 | 0 | 0 | 0 | 0 | 0 | 1 | 1 | 0 | 0 | 0 | 0 | -5 | -5 | 2 | 2 | 0 | 0 | 0 | 0 | 0 | 0 |
| Chai et al. (2017) | 0 | 0 | 0 | 0 | 0 | 0 | 0 | 0 | -3 | 3 | 0 | 0 | 0 | 0 | 0 | 0 | 1 | 1 | 0 | 0 | 0 | 0 | 2 | 2 | 2 | 2 | 2 | 2 | 0 | 0 | 0 | 1 |
| Elmohr et al. (2019) | 1 | 0 | 1 | 1 | 0 | 0 | 0 | 0 | 3 | 3 | 0 | 0 | 0 | 0 | 0 | 0 | 1 | 2 | 0 | 0 | 0 | 0 | 2 | 2 | 2 | 2 | 2 | 2 | 0 | 0 | 0 | 0 |
| Ho et al. (2019) | 0 | 1 | 0 | 1 | 0 | 0 | 0 | 0 | 3 | -3 | 0 | 0 | 0 | 0 | 0 | 0 | 1 | 1 | 0 | 0 | 0 | 0 | -5 | -5 | 2 | 2 | 2 | 2 | 0 | 0 | 0 | 0 |
| Kong et al. (2022) | 1 | 1 | 1 | 1 | 0 | 0 | 0 | 0 | 3 | 3 | 1 | 1 | 0 | 0 | 0 | 0 | 1 | 1 | 1 | 1 | 7 | 7 | 5 | 5 | 2 | 2 | 2 | 2 | 1 | 1 | 0 | 0 |
| Koyuncu et al. (2019) | 0 | 0 | 0 | 0 | 0 | 0 | 0 | 0 | 3 | 3 | 0 | 0 | 0 | 0 | 0 | 1 | 1 | 1 | 0 | 0 | 0 | 0 | 2 | 2 | 0 | 0 | 0 | 2 | 0 | 0 | 1 | 1 |
| Li et al. (2018) | 1 | 1 | 0 | 0 | 0 | 0 | 0 | 0 | 3 | 3 | 0 | 0 | 0 | 0 | 1 | 0 | 1 | 1 | 0 | 0 | 0 | 0 | -5 | -5 | 2 | 2 | 0 | 2 | 0 | 0 | 0 | 0 |
| Liu et al. (2021) | 1 | 1 | 1 | 1 | 0 | 0 | 0 | 0 | 3 | 3 | 0 | 0 | 0 | 0 | 1 | 1 | 1 | 1 | 0 | 0 | 0 | 0 | 2 | 2 | 2 | 2 | 2 | 2 | 0 | 0 | 0 | 0 |
| Nakajo et al. (2017) | 1 | 1 | 0 | 0 | 0 | 0 | 0 | 0 | -3 | -3 | 0 | 0 | 0 | 0 | 1 | 0 | 1 | 1 | 0 | 0 | 0 | 0 | -5 | -5 | 0 | 0 | 2 | 2 | 0 | 0 | 0 | 0 |
| Moawad et al. (2021) | 1 | 1 | 1 | 1 | 0 | 0 | 0 | 0 | 3 | 3 | 0 | 0 | 0 | 0 | 0 | 0 | 1 | 1 | 0 | 0 | 0 | 0 | 2 | 2 | 2 | 2 | 2 | 2 | 0 | 0 | 0 | 0 |
| Rocha et al. (2018) | 1 | 1 | 1 | 1 | 0 | 0 | 0 | 0 | -3 | -3 | 0 | 0 | 0 | 0 | 1 | 1 | 1 | 2 | 0 | 0 | 0 | 0 | -5 | -5 | 0 | 2 | 0 | 0 | 0 | 0 | 0 | 0 |
| Romeo et al. (2018) | 0 | 1 | 0 | 0 | 0 | 0 | 0 | 0 | 3 | 3 | 0 | 0 | 0 | 0 | 0 | 0 | 2 | 1 | 0 | 0 | 0 | 0 | 2 | 2 | 2 | 2 | 2 | 2 | 0 | 0 | 0 | 0 |
| Schieda et al. (2017) | 1 | 1 | 0 | 0 | 0 | 0 | 0 | 0 | -3 | -3 | 1 | 0 | 0 | 0 | 0 | 0 | 1 | 1 | 0 | 0 | 0 | 0 | -5 | -5 | 0 | 2 | 2 | 2 | 0 | 0 | 0 | 0 |
| Shi et al. (2019) | 1 | 1 | 1 | 1 | 0 | 0 | 0 | 0 | 3 | 3 | 0 | 0 | 0 | 0 | 0 | 0 | 2 | 2 | 0 | 0 | 0 | 0 | 2 | 2 | 2 | 2 | 2 | 2 | 0 | 0 | 0 | 0 |
| Shoemaker et al. (2018) | 0 | 0 | 0 | 0 | 0 | 0 | 0 | 0 | 3 | 3 | 0 | 0 | 0 | 0 | 0 | 0 | 2 | 2 | 0 | 0 | 0 | 0 | 2 | 2 | 2 | 2 | 2 | 0 | 0 | 0 | 1 | 1 |
| Stanzione et al. (2021) | 1 | 0 | 1 | 1 | 0 | 0 | 0 | 0 | 3 | 3 | 0 | 0 | 0 | 0 | 0 | 0 | 1 | 1 | 0 | 0 | 0 | 0 | 2 | 2 | 2 | 2 | 2 | 2 | 0 | 0 | 0 | 0 |
| Szász et al. (2020) | 1 | 1 | 1 | 1 | 0 | 0 | 0 | 0 | -3 | -3 | 0 | 0 | 0 | 0 | 1 | 1 | 1 | 1 | 0 | 0 | 0 | 0 | -5 | -5 | 2 | 2 | 2 | 2 | 0 | 0 | 0 | 0 |
| Torresan et al. (2021) | 1 | 1 | 1 | 1 | 0 | 0 | 0 | 0 | -3 | -3 | 0 | 0 | 0 | 0 | 0 | 0 | 1 | 1 | 0 | 0 | 0 | 0 | -5 | -5 | 0 | 2 | 2 | 2 | 0 | 0 | 0 | 0 |
| Tu et al. (2018) | 1 | 1 | 0 | 0 | 0 | 0 | 0 | 0 | -3 | -3 | 0 | 0 | 0 | 0 | 1 | 0 | 1 | 1 | 0 | 0 | 0 | 0 | -5 | -5 | 0 | 0 | 0 | 0 | 0 | 0 | 0 | 0 |
| Tu et al. (2020) | 1 | 1 | 0 | 0 | 0 | 0 | 0 | 0 | -3 | -3 | 0 | 0 | 0 | 0 | 0 | 0 | 1 | 1 | 0 | 0 | 0 | 0 | 2 | 2 | 2 | 2 | 2 | 2 | 0 | 0 | 0 | 0 |
| Tüdös et al. (2019) | 1 | 1 | 1 | 1 | 0 | 0 | 0 | 0 | -3 | -3 | 0 | 0 | 0 | 0 | 0 | 1 | 1 | 1 | 0 | 0 | 0 | 0 | -5 | -5 | 0 | 2 | 2 | 2 | 0 | 0 | 0 | 0 |
| Umanodan et al. (2017) | 1 | 1 | 1 | 1 | 0 | 0 | 0 | 0 | -3 | -3 | 0 | 0 | 0 | 0 | 1 | 1 | 1 | 1 | 0 | 0 | 0 | 0 | -5 | -5 | 2 | 2 | 2 | 2 | 0 | 0 | 0 | 0 |
| Wu et al. (2020) | 1 | 1 | 1 | 1 | 0 | 0 | 0 | 0 | -3 | -3 | 0 | 0 | 0 | 0 | 1 | 1 | 1 | 1 | 0 | 0 | 0 | 0 | -5 | -5 | 2 | 2 | 2 | 2 | 0 | 0 | 0 | 0 |
| Yi et al. (2018) | 1 | 1 | 1 | 1 | 0 | 0 | 0 | 0 | 3 | 3 | 0 | 0 | 0 | 0 | 0 | 0 | 1 | 1 | 0 | 0 | 0 | 0 | -5 | -5 | 2 | 2 | 2 | 2 | 0 | 0 | 0 | 0 |
| Yi et al. (2018)(2) | 1 | 1 | 1 | 1 | 0 | 0 | 0 | 0 | 3 | 3 | 0 | 0 | 0 | 0 | 1 | 1 | 1 | 1 | 1 | 1 | 0 | 0 | 2 | 2 | 2 | 2 | 2 | 2 | 0 | 0 | 0 | 0 |
| Yu et al. (2020) | 1 | 1 | 0 | 0 | 0 | 0 | 0 | 0 | -3 | -3 | 0 | 0 | 0 | 0 | 1 | 1 | 1 | 1 | 0 | 0 | 0 | 0 | -5 | -5 | 2 | 2 | 2 | 2 | 0 | 0 | 0 | 0 |
| Zhang et al. (2017) | 1 | 1 | 0 | 0 | 0 | 0 | 0 | 0 | -3 | -3 | 0 | 0 | 0 | 0 | 1 | 1 | 1 | 1 | 0 | 0 | 0 | 0 | -5 | -5 | 2 | 2 | 2 | 2 | 0 | 0 | 0 | 0 |
| Zheng et al. (2020) | 1 | 1 | 1 | 1 | 0 | 0 | 0 | 0 | 3 | 3 | 1 | 1 | 0 | 0 | 0 | 0 | 1 | 1 | 0 | 0 | 0 | 0 | 2 | 2 | 2 | 2 | 2 | 2 | 0 | 0 | 0 | 0 |
| Defined range | 0 – 2 | | 0 – 1 | | 0 – 1 | | 0 – 1 | | -3 – 3 | | 0 – 1 | | 0 – 1 | | 0–1 | | 0 – 2 | | 0 – 1 | | 0 – 7 | | -5 – 5 | | 0 – 2 | | 0 – 2 | | 0 – 1 | | 0 – 4 | |

| Table S3. RQS Ratings and average rating per item | | | | | | | | | | | | | | | | |  |
| --- | --- | --- | --- | --- | --- | --- | --- | --- | --- | --- | --- | --- | --- | --- | --- | --- | --- |
| Study ID | *Image Protocol* | *Multiple Segmentations* | *Phantom Study* | *Multiple Time Points* | *Feature Reduction* | *Non Radiomics* | *Biological Correlates* | *Cut-off* | *Discrimination and Resampling* | *Calibration* | *Prospective* | *Validation* | *Gold Standard* | *Clinical Utility* | *Cost-effectiveness* | *Open Science* | *Total* |
| Andersen et al. (2021) | 1 | 0 | 0 | 0 | 3 | 0 | 0 | 0 | 1 | 0 | 0 | -5 | 2 | 0 | 0 | 0 | 2 |
| Chai et al. (2017) | 0 | 0 | 0 | 0 | 3 | 0 | 0 | 0 | 1 | 0 | 0 | 2 | 2 | 2 | 0 | 1 | 11 |
| Elmohr et al. (2019) | 1 | 1 | 0 | 0 | 3 | 0 | 0 | 0 | 2 | 0 | 0 | 2 | 2 | 2 | 0 | 0 | 13 |
| Ho et al. (2019) | 1 | 0 | 0 | 0 | 3 | 0 | 0 | 0 | 1 | 0 | 0 | -5 | 2 | 2 | 0 | 0 | 4 |
| Kong et al. (2022) | 1 | 1 | 0 | 0 | 3 | 1 | 0 | 0 | 1 | 1 | 7 | 5 | 2 | 2 | 1 | 0 | 25 |
| Koyuncu et al. (2019) | 0 | 0 | 0 | 0 | 3 | 0 | 0 | 0 | 1 | 0 | 0 | 2 | 0 | 2 | 0 | 1 | 9 |
| Li et al. (2018) | 1 | 0 | 0 | 0 | 3 | 0 | 0 | 0 | 1 | 0 | 0 | -5 | 2 | 0 | 0 | 0 | 2 |
| Liu et al. (2021) | 1 | 1 | 0 | 0 | 3 | 0 | 0 | 1 | 1 | 0 | 0 | 2 | 2 | 2 | 0 | 0 | 13 |
| Nakajo et al. (2017) | 1 | 0 | 0 | 0 | -3 | 0 | 0 | 1 | 1 | 0 | 0 | -5 | 0 | 2 | 0 | 0 | -3 |
| Moawad et al. (2021) | 1 | 1 | 0 | 0 | 3 | 0 | 0 | 0 | 1 | 0 | 0 | 2 | 2 | 2 | 0 | 0 | 12 |
| Rocha et al. (2018) | 1 | 1 | 0 | 0 | -3 | 0 | 0 | 1 | 1 | 0 | 0 | -5 | 0 | 0 | 0 | 0 | -4 |
| Romeo et al. (2018) | 1 | 0 | 0 | 0 | 3 | 0 | 0 | 0 | 2 | 0 | 0 | 2 | 2 | 2 | 0 | 0 | 12 |
| Schieda et al. (2017) | 1 | 0 | 0 | 0 | -3 | 1 | 0 | 0 | 1 | 0 | 0 | -5 | 0 | 2 | 0 | 0 | -3 |
| Shi et al. (2019) | 1 | 1 | 0 | 0 | 3 | 0 | 0 | 0 | 2 | 0 | 0 | 2 | 0 | 2 | 0 | 0 | 11 |
| Shoemaker et al. (2018) | 0 | 0 | 0 | 0 | 3 | 0 | 0 | 0 | 2 | 0 | 0 | 2 | 2 | 2 | 0 | 1 | 12 |
| Stanzione et al. (2021) | 1 | 1 | 0 | 0 | 3 | 0 | 0 | 0 | 1 | 0 | 0 | 2 | 0 | 2 | 0 | 0 | 10 |
| Szász et al. (2020) | 1 | 1 | 0 | 0 | -3 | 0 | 0 | 1 | 1 | 0 | 0 | -5 | 2 | 2 | 0 | 0 | 0 |
| Torresan et al. (2021) | 1 | 1 | 0 | 0 | -3 | 0 | 0 | 0 | 1 | 0 | 0 | -5 | 0 | 2 | 0 | 0 | -3 |
| Tu et al. (2018) | 1 | 0 | 0 | 0 | -3 | 0 | 0 | 1 | 1 | 0 | 0 | -5 | 0 | 0 | 0 | 0 | -5 |
| Tu et al. (2020) | 1 | 0 | 0 | 0 | -3 | 0 | 0 | 0 | 1 | 0 | 0 | 2 | 0 | 2 | 0 | 0 | 3 |
| Tüdös et al. (2019) | 1 | 1 | 0 | 0 | -3 | 0 | 0 | 1 | 1 | 0 | 0 | -5 | 0 | 2 | 0 | 0 | -2 |
| Umanodan et al. (2017) | 1 | 1 | 0 | 0 | -3 | 0 | 0 | 1 | 1 | 0 | 0 | -5 | 0 | 2 | 0 | 0 | -2 |
| Wu et al. (2020) | 1 | 1 | 0 | 0 | -3 | 0 | 0 | 1 | 1 | 0 | 0 | -5 | 0 | 2 | 0 | 0 | -2 |
| Yi et al. (2018) | 1 | 1 | 0 | 0 | 3 | 0 | 0 | 0 | 1 | 0 | 0 | -5 | 2 | 2 | 0 | 0 | 5 |
| Yi et al. (2018)(2) | 1 | 1 | 0 | 0 | 3 | 0 | 0 | 1 | 1 | 1 | 0 | 2 | 2 | 2 | 0 | 0 | 14 |
| Yu et al. (2020) | 1 | 0 | 0 | 0 | -3 | 0 | 0 | 1 | 1 | 0 | 0 | -5 | 0 | 2 | 0 | 0 | -3 |
| Zhang et al. (2017) | 1 | 0 | 0 | 0 | -3 | 0 | 0 | 1 | 1 | 0 | 0 | -5 | 2 | 2 | 0 | 0 | -1 |
| Zheng et al. (2020) | 1 | 1 | 0 | 0 | 3 | 1 | 0 | 0 | 1 | 0 | 0 | 2 | 2 | 2 | 0 | 0 | 13 |
| Mode | 1 | 1 | 0 | 0 | 3 | 0 | 0 | 0 | 1 | 0 | 0 | -5 | 2 | 2 | 0 | 0 |  |
| Defined range | 0 – 2 | 0 – 1 | 0 – 1 | 0 – 1 | -3 – 3 | 0 – 1 | 0 – 1 | 0 – 1 | 0 – 2 | 0 – 1 | 0 – 7 | -5 – 5 | 0 – 2 | 0 – 2 | 0 – 1 | 0 – 4 |  |

| Table S4. Individual QUADAS-2 Ratings (HZ / HL) | | | | | | | | | | | | | | |
| --- | --- | --- | --- | --- | --- | --- | --- | --- | --- | --- | --- | --- | --- | --- |
| Study | Risk of Bias | | | | | | | | Applicability Concerns | | | | | |
|  | Patient  Selection | | Index  Test | | Reference  Standard | | Flow and  Timing | | Patient  Selection | | Index  Test | | Reference  Standard | |
| Andersen et al. (2021) | Low | Low | Low | Low | Low | Low | Unclear | Unclear | Low | Low | Low | Low | Low | Low |
| Chai et al. (2017) | High | High | High | High | Low | Low | Unclear | Unclear | High | Low | Low | Low | Low | Low |
| Elmohr et al. (2019) | Low | Low | Low | Low | Low | Low | Unclear | Unclear | Low | Low | Low | Low | Low | Low |
| Ho et al. (2019) | High | High | High | High | High | High | Unclear | Unclear | Low | High | Low | Low | High | High |
| Kong et al. (2022) | High | High | High | High | Low | Low | Unclear | Unclear | Low | Low | Low | Low | Low | Low |
| Koyuncu et al. (2019) | High | High | High | High | Unclear | Unclear | Unclear | Unclear | High | High | Low | High | Unclear | Unclear |
| Li et al. (2018) | High | High | High | High | Low | Low | Unclear | Unclear | Low | Low | Low | Low | Low | Low |
| Liu et al. (2021) | High | Low | Low | Low | Low | Low | Unclear | Unclear | Low | Low | Low | Low | Low | Low |
| Nakajo et al. (2017) | Low | High | Low | Low | High | High | Unclear | Unclear | Low | Low | Low | Low | High | High |
| Moawad et al. (2021) | Low | Low | High | High | Low | Low | Unclear | Unclear | Low | Low | Low | Low | Low | Low |
| Rocha et al. (2018) | High | High | Low | Low | Low | Low | Unclear | Unclear | Low | Low | Low | Low | Low | Low |
| Romeo et al. (2018) | Low | Low | High | High | High | High | Unclear | Unclear | Low | Low | Low | Low | High | High |
| Schieda et al. (2017) | Low | Low | Low | Low | High | High | Unclear | Unclear | Low | Low | Low | Low | High | High |
| Shi et al. (2019) | Low | Low | Low | Low | Low | Low | Unclear | Unclear | Low | Low | Low | Low | Low | Low |
| Shoemaker et al. (2018) | High | High | High | Unclear | Low | Low | Unclear | Unclear | High | High | Low | Low | Low | Low |
| Stanzione et al. (2021) | Low | Low | High | High | High | High | Unclear | Unclear | Low | Low | Low | Low | High | High |
| Szász et al. (2020) | High | High | Low | Low | Low | Low | Unclear | Unclear | Low | Low | Low | Low | Low | Low |
| Torresan et al. (2021) | High | High | High | High | Low | Low | Unclear | Unclear | Low | Low | Low | Low | Low | Low |
| Tu et al. (2018) | Low | Low | Low | Low | High | High | Unclear | Unclear | Low | Low | Low | Low | High | High |
| Tu et al. (2020) | High | High | Low | Low | High | High | Unclear | Unclear | Low | High | Low | Low | High | High |
| Tüdös et al. (2019) | High | High | Low | Low | High | High | Unclear | Unclear | Low | Low | Low | Low | High | High |
| Umanodan et al. (2017) | Low | Low | Low | Low | High | High | Unclear | Unclear | Low | Low | Low | Low | High | High |
| Wu et al. (2020) | High | High | Low | Low | Low | Low | Unclear | Unclear | Low | Low | Low | Low | Low | Low |
| Yi et al. (2018) | High | High | Low | Low | Low | Low | Unclear | Unclear | Low | Low | Low | Low | Low | Low |
| Yi et al. (2018)(2) | Low | Low | Low | Low | Low | Low | Unclear | Unclear | Low | Low | Low | Low | Low | Low |
| Yu et al. (2020) | High | High | Low | Low | Low | Low | Unclear | Unclear | Low | Low | Low | Low | Low | Low |
| Zhang et al. (2017) | High | Low | Low | Low | Low | Low | Unclear | Unclear | Low | Low | Low | Low | Low | Low |
| Zheng et al. (2020) | Low | Low | High | High | Low | Low | Unclear | Unclear | Low | Low | High | High | Low | Low |
| Low = Low risk; High = High risk; Unclear = Unclear risk. | | | | | | | | | | | | | | |

| Table S5. Result of QUADAS-2 rating. | | | | | | | |
| --- | --- | --- | --- | --- | --- | --- | --- |
| Study | Risk of Bias | | | | Applicability Concerns | | |
|  | Patient  Selection | Index  Test | Reference  Standard | Flow and  Timing | Patient  Selection | Index  Test | Reference  Standard |
| Andersen et al. (2021) | Low | Low | Low | Unclear | Low | Low | Low |
| Chai et al. (2017) | High | High | Low | Unclear | Low | Low | Low |
| Elmohr et al. (2019) | Low | Low | Low | Unclear | Low | Low | Low |
| Ho et al. (2019) | High | High | High | Unclear | Low | Low | High |
| Kong et al. (2022) | High | High | Low | Unclear | Low | Low | Low |
| Koyuncu et al. (2019) | High | High | Unclear | Unclear | High | Low | Unclear |
| Li et al. (2018) | High | High | Low | Unclear | Low | Low | Low |
| Liu et al. (2021) | High | Low | Low | Unclear | Low | Low | Low |
| Nakajo et al. (2017) | High | Low | High | Unclear | Low | Low | High |
| Moawad et al. (2021) | Low | High | Low | Unclear | Low | Low | Low |
| Rocha et al. (2018) | High | Low | Low | Unclear | Low | Low | Low |
| Romeo et al. (2018) | Low | High | High | Unclear | Low | Low | High |
| Schieda et al. (2017) | Low | Low | High | Unclear | Low | Low | High |
| Shi et al. (2019) | Low | Low | Low | Unclear | Low | Low | Low |
| Shoemaker et al. (2018) | High | Unclear | Low | Unclear | High | Low | Low |
| Stanzione et al. (2021) | Low | High | High | Unclear | Low | Low | High |
| Szász et al. (2020) | High | Low | Low | Unclear | Low | Low | Low |
| Torresan et al. (2021) | High | High | Low | Unclear | Low | Low | Low |
| Tu et al. (2018) | Low | Low | High | Unclear | Low | Low | High |
| Tu et al. (2020) | High | Low | High | Unclear | Low | Low | High |
| Tüdös et al. (2019) | High | Low | High | Unclear | Low | Low | High |
| Umanodan et al. (2017) | Low | Low | High | Unclear | Low | Low | High |
| Wu et al. (2020) | High | Low | Low | Unclear | Low | Low | Low |
| Yi et al. (2018) | High | Low | Low | Unclear | Low | Low | Low |
| Yi et al. (2018)(2) | Low | Low | Low | Unclear | Low | Low | Low |
| Yu et al. (2020) | High | Low | Low | Unclear | Low | Low | Low |
| Zhang et al. (2017) | High | Low | Low | Unclear | Low | Low | Low |
| Zheng et al. (2020) | Low | High | Low | Unclear | Low | High | Low |
| Low = Low risk; High = High risk; Unclear = Unclear risk. | | | | | | | |

| Table S6. The results of sensitivity analyses for each study | | | | | | | |
| --- | --- | --- | --- | --- | --- | --- | --- |
| Eliminated study | Sensitivity | Specificity | PLR | NLR | DOR | AUC | |
| Andersen et al. (2021) | 0.82(0.71-0.90) | 0.84(0.72-0.92) | 5.26(2.78-9.97) | 0.21(0.13-0.36) | 24.67(9.31-65.40) | 0.90(0.87-0.92) |  |
| Koyuncu et al. (2019) | 0.80(0.67-0.89) | 0.84(0.71-0.92) | 4.93(2.58-9.42) | 0.24(0.14-0.42) | 20.60(7.35-57.77) | 0.89(0.86-0.91) |  |
| Li et al. (2018) | 0.75(0.64-0.83) | 0.85(0.75-0.92) | 5.08(2.72-9.47) | 0.29(0.19-0.45) | 17.31(6.41-46.72) | 0.87(0.84-0.90) |  |
| Moawad et al. (2021) | 0.79(0.66-0.88) | 0.85(0.73-0.92) | 5.13(2.71-9.70) | 0.25(0.14-0.43) | 20.64(7.43-57.36) | 0.89(0.86-0.91) |  |
| Rocha et al. (2018) | 0.78(0.65-0.87) | 0.81(0.71-0.88) | 4.10(2.59-6.48) | 0.27(0.16-0.46) | 15.18(6.54-35.21) | 0.87(0.83-0.89) |  |
| Shi et al. (2019) | 0.80(0.67-0.89) | 0.85(0.72-0.92) | 5.18(2.68-10.03) | 0.24(0.13-0.42) | 21.93(7.63-63.06) | 0.89(0.86-0.92) |  |
| Torresan et al. (2021) | 0.79(0.66-0.88) | 0.82(0.71-0.90) | 4.50(2.61-7.75) | 0.26(0.15-0.43) | 17.55(7.03-43.82) | 0.88(0.85-0.90) |  |
| Tu et al. (2018) | 0.83(0.73-0.89) | 0.84(0.72-0.92) | 5.28(2.85-9.77) | 0.21(0.13-0.33) | 25.49(10.35-62.79) | 0.90(0.87-0.92) |  |
| Yu et al. (2020) | 0.79(0.65-0.88) | 0.76(0.72-0.80) | 3.36(2.81-4.01) | 0.27(0.16-0.47) | 12.29(6.52-23.19) | 0.80(0.76-0.83) |  |
| Overall | 0.80(0.68-0.88) | 0.83(0.73-0.90) | 4.70(2.80-8.00) | 0.25(0.15-0.41) | 19.06(7.87-46.19) | 0.88(0.85-0.91) |  |
| PLR: positive likelihood ratio; NLR: negative likelihood ratio; DOR: diagnostic odds ratio; AUC: area under the curve.  The 95% confidence intervals are shown in parentheses. | | | | | | |  |
