# Supplementary figures and images for: Diagnostic performance of radiomics in adrenal masses: A systematic review and meta-analysis
Source: Front Oncol. 2022 Sep 2;12:975183. doi: 10.3389/fonc.2022.975183 (PMC9478189; doi:10.3389/fonc.2022.975183)

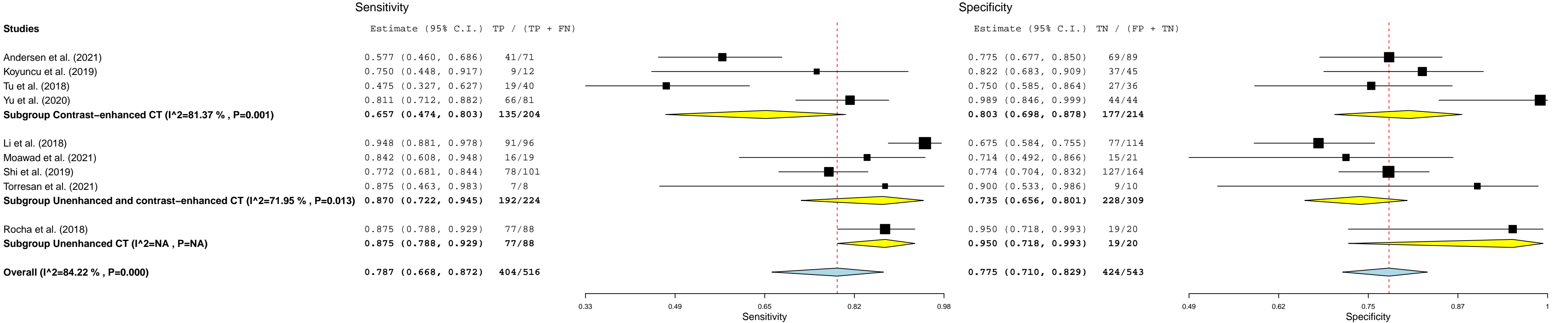

Supplement: Supplementary file 2 [file DataSheet_1.zip › Supplementary Figure/Figure S1.pdf]

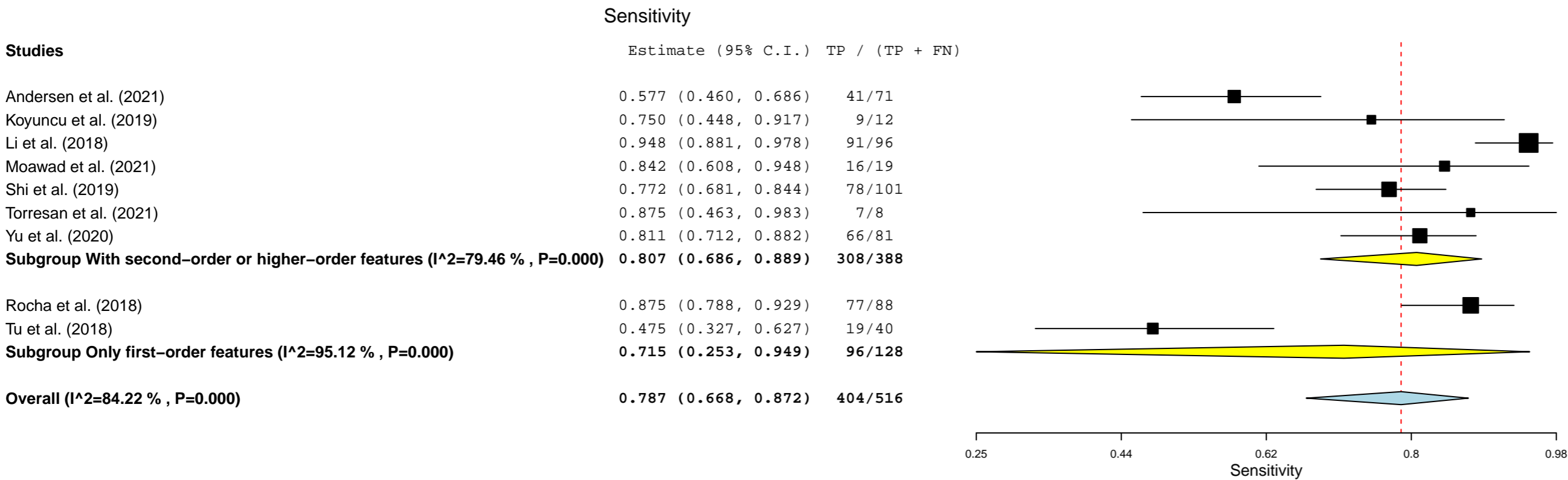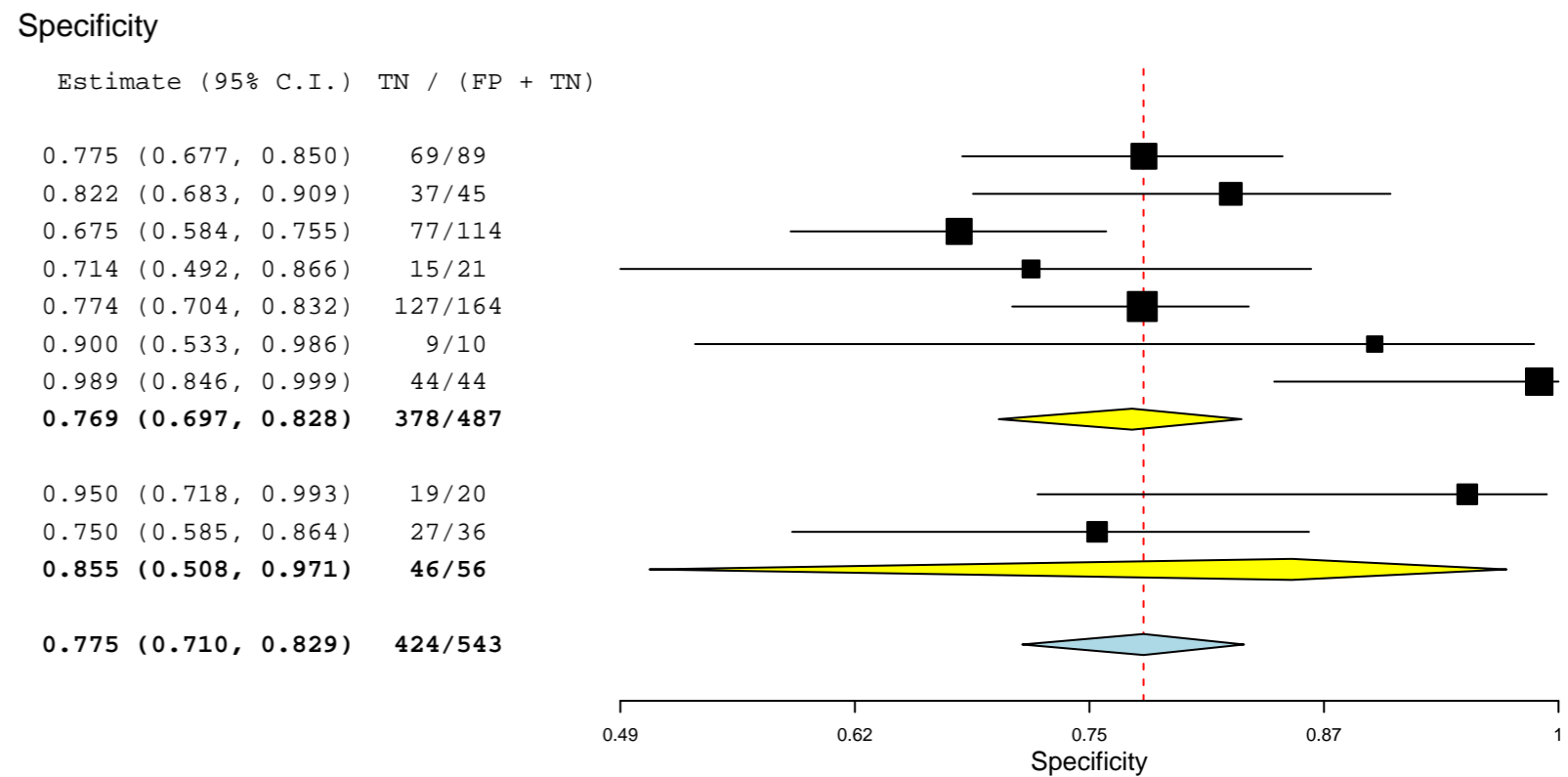

Supplement: Supplementary file 2 [file DataSheet_1.zip › Supplementary Figure/Figure S2.pdf]

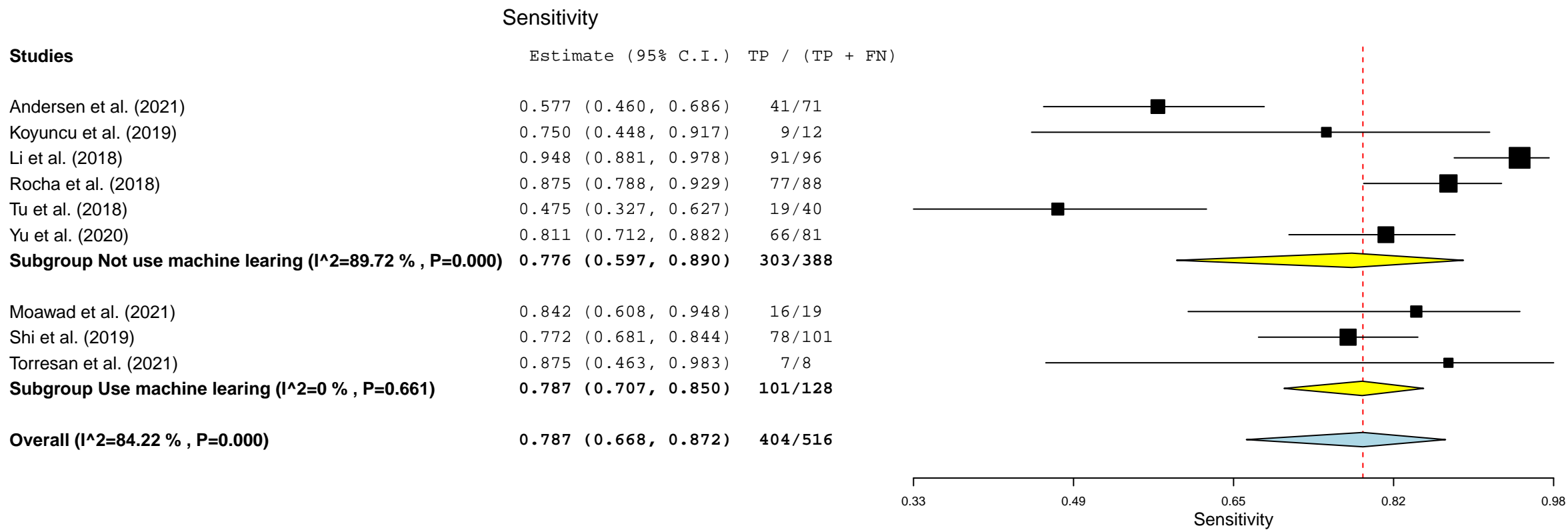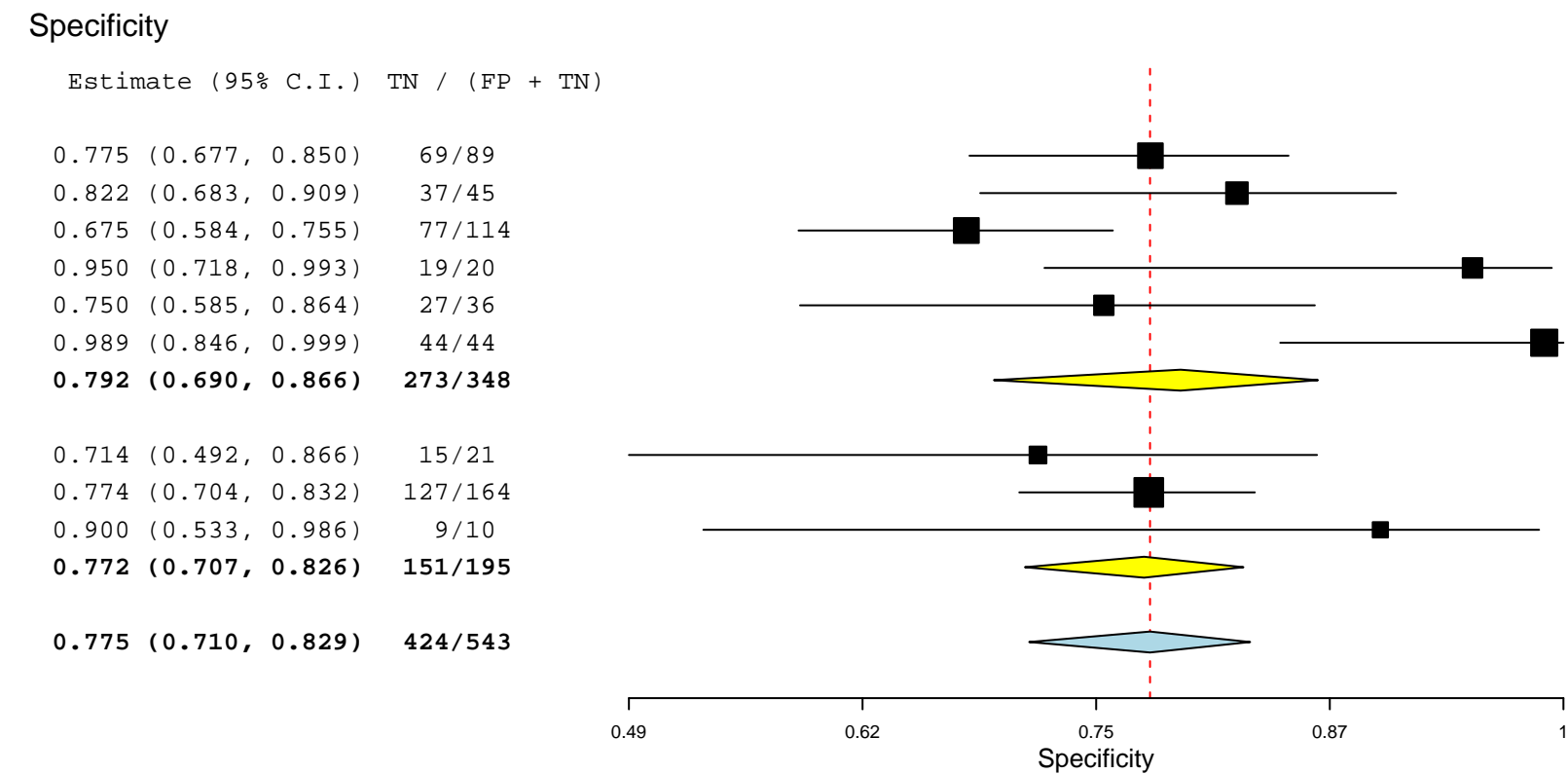

Supplement: Supplementary file 2 [file DataSheet_1.zip › Supplementary Figure/Figure S3.pdf]

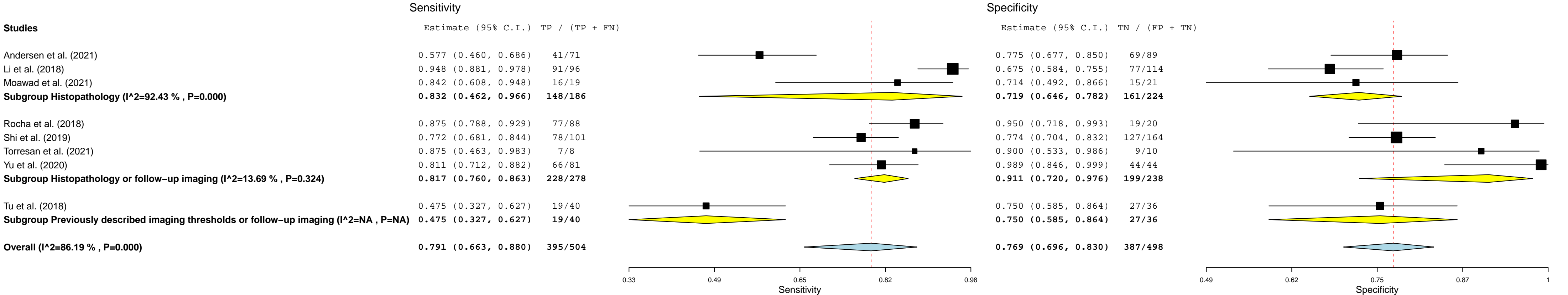

Supplement: Supplementary file 2 [file DataSheet_1.zip › Supplementary Figure/Figure S4.pdf]

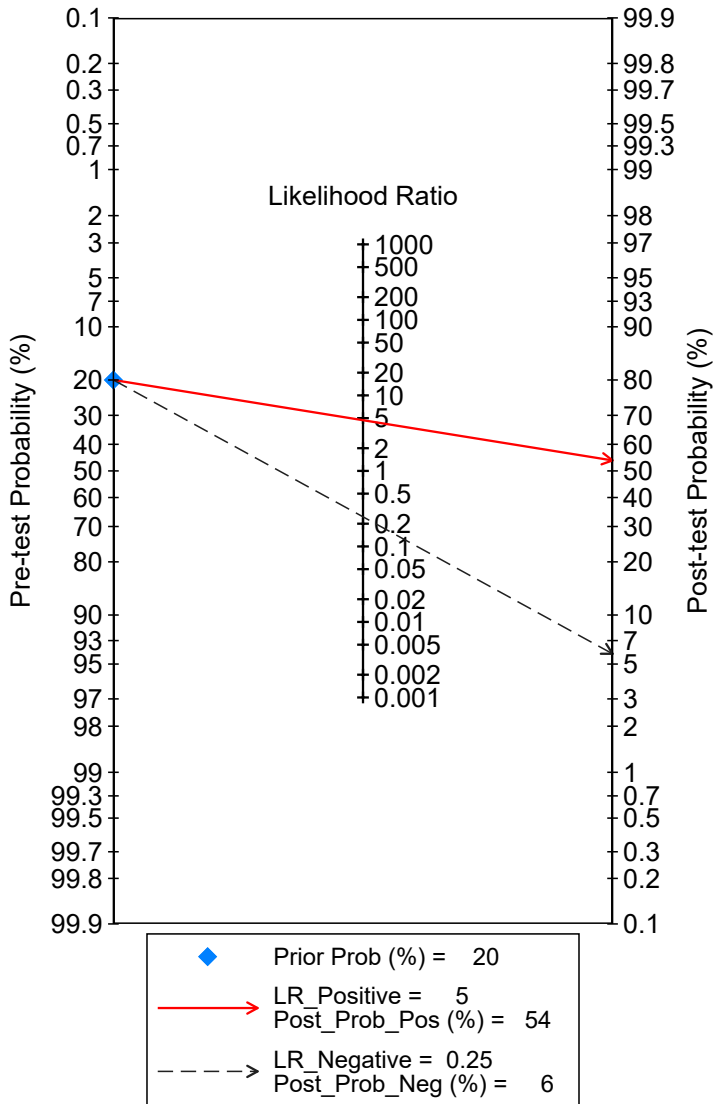

Supplement: Supplementary file 2 [file DataSheet_1.zip › Supplementary Figure/Figure S5.pdf]
